# Direct CVD Growth of Graphene on Technologically Important Dielectric and Semiconducting Substrates

**DOI:** 10.1002/advs.201800050

**Published:** 2018-09-22

**Authors:** Afzal Khan, Sk Masiul Islam, Shahzad Ahmed, Rishi R. Kumar, Mohammad R. Habib, Kun Huang, Ming Hu, Xuegong Yu, Deren Yang

**Affiliations:** ^1^ State Key Laboratory of Silicon Materials and School of Materials Science and Engineering Zhejiang University Hangzhou Zhejiang 310027 China; ^2^ Optoelectronics and MOEMS Group Council of Scientific and Industrial Research‐Central Electronics Engineering Research Institute Pilani 333031 Rajasthan India; ^3^ Academy of Scientific and Innovative Research (AcSIR) Ghaziabad 201002 Uttar Pradesh India; ^4^ Centre for Nanoscience and Nanotechnology Jamia Millia Islamia (Central University) New Delhi 110025 India; ^5^ State Key Laboratory of Silicon Materials and College of Information Science and Electronic Engineering Zhejiang University Hangzhou Zhejiang 310027 China

**Keywords:** catalyst free, dielectrics, direct chemical vapor deposition (CVD) growth, graphene, semiconductors

## Abstract

To fabricate graphene based electronic and optoelectronic devices, it is highly desirable to develop a variety of metal‐catalyst free chemical vapor deposition (CVD) techniques for direct synthesis of graphene on dielectric and semiconducting substrates. This will help to avoid metallic impurities, high costs, time consuming processes, and defect‐inducing graphene transfer processes. Direct CVD growth of graphene on dielectric substrates is usually difficult to accomplish due to their low surface energy. However, a low‐temperature plasma enhanced CVD technique could help to solve this problem. Here, the recent progress of metal‐catalyst free direct CVD growth of graphene on technologically important dielectric (SiO_2_, ZrO_2_, HfO_2_, h‐BN, Al_2_O_3_, Si_3_N_4,_ quartz, MgO, SrTiO_3,_ TiO_2_, etc.) and semiconducting (Si, Ge, GaN, and SiC) substrates is reviewed. High and low temperature direct CVD growth of graphene on these substrates including growth mechanism and morphology is discussed. Detailed discussions are also presented for Si and Ge substrates, which are necessary for next generation graphene/Si/Ge based hybrid electronic devices. Finally, the technology development of the metal‐catalyst free direct CVD growth of graphene on these substrates is concluded, with future outlooks.

## 1 Introduction

Nowadays, graphene has attracted tremendous research interest due to its extraordinary properties, such as high optical transparency, good electrical and thermal conductivities, mechanical flexibility, high intrinsic carrier mobility, and chemical stability. Owing to its excellent characteristics, 2D graphene sheet is regarded as a next‐generation transparent conductive electrode for applications in various electronic devices.^1^ To fabricate next generation electronic devices incorporating graphene, it is pertinent to develop a variety of methods for direct synthesis of graphene on any substrate. However, various methods have been adopted for controlled growth of graphene, and it was found that direct growth of graphene especially on dielectrics is difficult to achieve due to their low surface energy.^2^, ^3^, ^4^, ^5^, ^6^, ^7^, ^8^, ^9^, ^10^, ^11^ However, the surface modification of dielectric substrates can facilitate the ease of nucleation of graphene.^10^, ^12^ A low‐temperature growth of graphene on dielectric substrates can be achieved by stimulating the decomposition of the gaseous carbon source through plasma enhanced chemical vapor deposition (PECVD) technique.^4^, ^5^, ^12^ Furthermore, it is cumbersome to control the graphene growth rate and nucleation density on dielectric substrates compared to growth on metallic substrates. Generally, polymer assisted transfer and metal etching processes are employed to transfer the metal‐catalyzed chemical vapor deposition (CVD) grown graphene films on dielectric and semiconducting substrates. The metal‐catalyst free direct growth of graphene via CVD techniques on dielectric and semiconducting substrates is highly desirable to avoid metallic impurities during fabrication of electronic devices. This will also help in avoiding costly, time consuming, and defect inducing transfer process. Moreover, graphene/semiconductor hybrid structures especially graphene/Si and graphene/Ge seem to be very promising candidates for future transistors because of the adjustable Schottky barrier (SB) which forms between graphene/semiconductor. However, SB between graphene and a hydrogen‐terminated semiconductor is different from a conventional SB in two distinct manners. First, the generation of interface states is reduced due to the negligible interaction between chemically inert graphene and entirely saturated (without dangling bonds) semiconductor surface. Second, the work function of graphene can be adjusted by tuning the Fermi energy (*E*_F_) over a wide range through the electrostatic field effect.^13^ Large single‐crystalline Si wafers are easily available for the epitaxial graphene growth. However, the weak carbon diffusivity on Si surface and strong carbon solubility at high temperature deteriorate the quality of graphene grown on Si.^14^ Ni and Cu are well known for catalyzing the growth of multilayered graphenes (MLG) and monolayer graphenes, respectively. At high temperature, carbon atoms are dissolved in the metal chain followed by segregation to form graphene on their surfaces during lowering of temperature. Ni has higher carbon solubility and provides a larger pool, whereas Cu provides a smaller pool due to the lower solubility. Therefore, multilayer and monolayer graphenes form on Ni and Cu surfaces, respectively. On the other hand, Si provides even lower carbon solubility compared to Cu. According to the phase diagram of Si‐C, a straight SiC line exists at high temperatures ranging from 1000–2545 ± 40 °C. This suggests that single graphene and Si phases do not grow at the same time in this temperature range. Hence, Si substrate temperature should be less than 1000 °C for graphene growth.^15^ Based on the above discussions, single‐crystalline Ge substrates seem to be better option for the metal‐catalyst free direct CVD growth of single‐crystalline monolayer graphene to fabricate graphene/semiconductor heterostructure. The resulting low energy barrier leads to catalytic decomposition of carbon precursor, and promotes the formation of graphitic carbon on the surface.^16^ On the contrary, it enables extremely low solubility for carbon even at its melting temperature (<108 atoms per cm^3^),^17^ which enables the growth of complete monolayer graphene.^18^ Merging of multiple seeds into a single‐crystal layer with no grain boundary is possible due to distinct and anisotropic atomic arrangement of single‐crystal Ge surface. Furthermore, epitaxially grown large‐area single‐crystalline Ge layers on Si wafers are easily available, whereas negligible difference in thermal expansion coefficients between Ge and graphene helps in lowering the intrinsic wrinkle formation.^19^, ^20^

**Figure 1:**
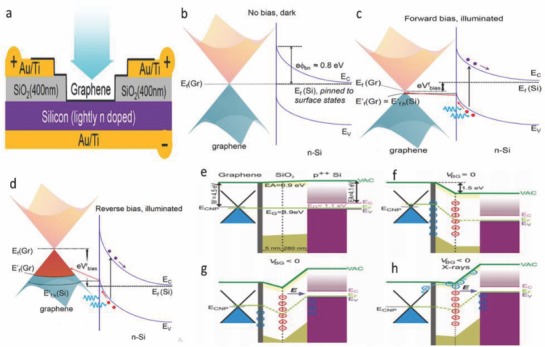
a) Schematic of graphene/Si heterojunction device during forward bias condition. b) Thermal equilibrium energy band diagram of the heterojunction in darkness. c) Application of a forward bias minimizes the number of accessible states for the injection of photoexcited holes from Si. d) Reverse bias results a large number of accessible states for photoexcited holes injected from Si under illumination. Reproduced with permission.^21^ Copyright 2013, American Chemical Society. e) Band alignment for the graphene based transistor. f) For the X‐ray irradiated device the built in positive charge induces n‐doping of graphene and the formation of a potential barrier. g) If the negative VBG is applied to the gate, the potential barrier is partially decreased and transformed into a potential well. h) The photoexcited electrons localized in the SiO_2_ conduction band will be drifted by an applied electric field and accumulate near the location of the positive charge. Reproduced with permission.^22^ Copyright 2017, Springer Nature.

CVD graphene on Si is a planar 2D heterojunction which forms a conventional Schottky‐diode‐like structure.^21^ This configuration can suitably construct a platform for optoelectronic device applications. In these devices, the photoexcitation takes place in Si, whereas graphene acts as a carrier collector. In addition, the Fermi‐levels of graphene can also be shifted with application of low reverse‐bias voltage despite the large amount of bias voltage required in capacitively coupled gates. Schematic of the device structure consisting of monolayer graphene/Si is shown in **Figure** **1**a.^21^ Energy band diagram pertaining to the Fermi levels of graphene (*E*_f_(Gr)) and lightly doped Si (*E*_f_(Si)) at thermal equilibrium (dark condition) is shown in Figure 1b. Figure 1c shows the condition at low forward bias *V* ^f^_bias_, which brings the Fermi level downward with respect to its “unbiased” condition. In this way, the Fermi level comes into close proximity to the quasi Fermi level for holes in Si, and thereby the number of accessible states for photoexcitation is significantly reduced. When reverse bias is applied, *E*_f_(Gr) shifts to higher value and generates a large number of available energy states for holes to inject (Figure 1d). Similarly, schematic band alignment (metal‐dielectric‐semiconductor) of the graphene based field effect transistor is shown in Figure 1e.^22^ The donor‐like defects *N*_D_^+^ ionized by X‐rays create a large potential barrier which prevents the electrons to cross graphene/SiO_2_ interface (Figure 1f). In addition, application of negative voltage creates a potential drop within the gate oxide resulting in decrease of the potential barrier along with transformation into a potential well (Figure 1g). The electron accumulation leads to gradual compensation of the positive charge and consequently lowering of the energy barrier (Figure 1h).^22^

As both transfer and fabrication processes of graphene always facilitate poor electrical and optical characteristics, therefore efforts have been made to improve the quality of patterned graphene due to the gradual demand of bandgap engineering and sub‐micrometer scale interconnections for high speed integrated circuits (ICs).^8^, ^9^, ^10^ Therefore, lack of suitable transfer process and the performance degradation caused by mechanical transfer of graphene imply that the metal‐catalyst free direct CVD growth of graphene on solid inorganic insulating and semiconducting substrates will be a niche area of research for graphene based electronics.

Through the above discussion, it is evident that these inventions pave a way toward the production of graphene, in‐depth knowledge of the correlation among products, microscopic processes, and experimental conditions. This motivates the research for the metal‐catalyst free direct CVD growth of graphene on various dielectric and semiconducting substrates. Herein, we present a comprehensive review focused on the recent progress made toward the metal‐catalyst free direct CVD growth of graphene on technologically important dielectric substrates such as SiO_2_, ZrO_2_, HfO_2_, h‐BN, Al_2_O_3_, Si_3_N_4_, quartz, MgO, SrTiO_3,_ TiO_2_, AlN, glass, and mica, and semiconducting substrates such as Si, Ge, GaN, and SiC. Merits and demerits of using high and low temperature CVD processes including growth mechanism and morphology of the graphene on these substrates have been discussed. Detailed discussions are presented for Si and Ge substrates, as they are important semiconductors, and suitable for next generation graphene/(Si/Ge) based hybrid electronic devices. Important results have been summarized in tables, and finally conclusions and outlook have been presented.

## 2 Catalyst‐Free Direct CVD Growth of Graphene on Technologically Important Dielectric Substrates

### 2.1 Graphene on SiO_2_ Substrates

**Figure 2:**
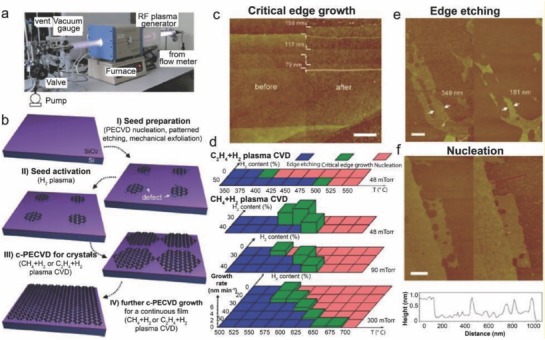
a) Photograph of the remote radio frequency PECVD technique used during the experiment. b) Schematic representation of the c‐PECVD technique. c) AFM images of a graphene flake being peeled‐off before (left) and after (right) c‐PECVD growth. d) Experimental data are plotted as a function of the temperature and H_2_ content at 48, 90, and 300 mTorr. The blue, green, and red colors indicate the parameters for edge etching, critical edge growth, and cluster nucleation, respectively. The height of the green columns denotes the growth rate. e,f) AFM images of peel‐off graphene flakes after activation of the edges with a H_2_ plasma (250 mTorr, 500 °C) for 20 min (left columns), followed by CH_4_+H_2_ plasma CVD (30% H_2_, 300 mTorr, 550 °C) for 80 min [(e), right column] or CH_4_+H_2_ plasma CVD (20% H_2_, 300 mTorr, 600 °C) for 40 min [(f), right column]. The profile of the height along the red line is shown below the AFM image (f). Scale bars for (c,e,f) are maintained at 500 nm. Reproduced with permission.^26^ Copyright 2013, Wiley‐VCH.

A low temperature growth leads to compatibility and minimizes energy consumption along with cost effectiveness for bulk production in the industry. Therefore, in order to deposit a clean high‐quality graphene directly on dielectric substrates, a controllable, low‐cost, and reliable mode is important at low temperature. The catalyst‐free direct growth of polycrystalline graphene was achieved by the pyrolysis of methane (CH_4_) on bare SiO_2_/Si substrates via oxygen aided atmospheric pressure chemical vapor deposition (APCVD) process.^23^ However, a low‐temperature (550–650 °C) growth of graphene on SiO_2_ substrates could be achieved by using PECVD.^24^, ^25^, ^26^ A PECVD system with 80 W power and 13.56 mHz radio frequency is shown in **Figure** **2**a. The SiO_2_/Si substrate was cleaned and then heated in H_2_ (99.9995%; 50 mTorr) atmosphere at 1000 °C for 15 min. Three kinds of seeds were prepared onto the substrates, which include 1) mechanical exfoliation using scotch tape peel‐off graphene; 2) graphitic clusters nucleated by C_2_H_4_ (99.9%) + H_2_ plasma CVD (50% H_2_, 48 mTorr, 550 °C) or CH_4_ (99.9%) + H_2_ plasma CVD (30% H_2_, 48 mTorr, 650 °C); and 3) patterning of nanoislands graphene by oxygen plasma etching and electron beam lithography. The position of the substrate with the seeds was at the center of the furnace. H_2_ plasma (H_2_: 250 mTorr) was generated upstream to activate the edge of the seeds at 500 °C. C_2_H_4_+H_2_ plasma CVD (50% H_2_, 48 mTorr, 500 °C) or CH_4_+H_2_ plasma CVD (30% H_2_, 48 mTorr, 600 °C) was then used for graphene growth. The PECVD growth mechanism of graphene on SiO_2_/Si substrate is shown in Figure 2b. Before and after PECVD growth, a trilayer peeled‐off graphene flake was observed in atomic force microscopy (AFM) images (Figure 2c). The movement of the edges, i.e., upper, middle, and bottom layer was found to be 158, 117, and 79 nm, respectively. This gave the indication that flake growth occurred at the edges continuously instead of in the plane. The critical parameters, such as H_2_ content, the pressure, and the growth temperature decided the growth at the edge (Figure 2d). At a lower temperature of 550 °C, the edges of the flakes were etched about 168 nm (Figure 2e). Small graphitic clusters were nucleated on the entire surface of the graphene flakes instead of the edge growth at a lower H_2_ content during CH_4_+H_2_ plasma CVD treatment, whereas SiO_2_/Si surface having heights less than 1 nm was found (Figure 2f). The heights observed in the images revealed the single‐layered nature of the clusters. Growth took place only at the edges at a well‐controlled critical temperature (Figure 2c). This critical temperature decreased with decreasing H_2_ concentration (Figure 2d). For edge growth, the critical temperature decreased to as low as 400 °C when C_2_H_4_ was used as the source of carbon in c‐PECVD (0% H_2_, 48 mTorr) (Figure 2d). Moreover, growth rate can be improved at low pressure. At 250 mTorr, the growth rate (30% H_2_, 600 °C) was found 1 nm min^−1^ and increased about 4.5 nm min^−1^ at 48 mTorr.

In addition to this, the metal‐catalyst free direct CVD growth of graphene on SiO_2_ has been reported by many researchers. Wang et al.^27^ studied the growth of vertically aligned graphene nanosheets (VAGNs) on SiO_2_ substrate using CH_4_ as a precursor via thermal APCVD. Chen et al.^28^ reported single crystal hexagonal and dodecagonal patterns on SiO_2_ substrate using CH_4_ as a precursor via near equilibrium CVD. Few‐layer graphene films on SiO_2_ substrate using CH_4_ as a precursor via APCVD were demonstrated by Bi et al.,^29^ whereas Zhao et al.^30^ investigated the graphene nanowalls on SiO_2_ substrate using CH_4_ as a precursor via PECVD technique. It was observed that a low temperature (400 °C) direct growth of micrometer‐scale graphene crystals on SiO_2_ substrates could be achieved by using PECVD technique. These graphene crystals can be directly embedded to fabricate electronic devices, thereby eliminating the conventional postdeposition transfer process. Lack of transfer process, good control of the method, excellent quality of grown graphene, and the compatibility of this process with the current microelectronics technology make it a facile approach for future use in graphene electronics.

### 2.2 Graphene on ZrO_2_ Substrates

**Figure 3:**
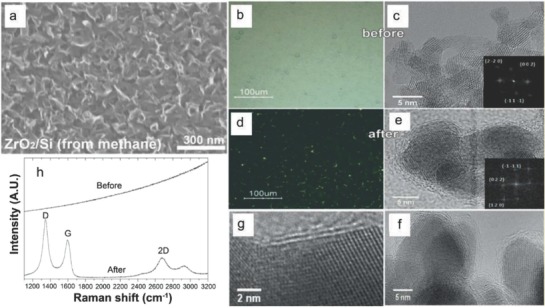
a) SEM images of VGs on ZrO_2_/Si substrates. The VGs were grown at a condition having 8 sccm CH_4_, 50 sccm H_2_, and 50 sccm Ar for 6 h. Reproduced with permission.^27^ Copyright 2017, Elsevier Ltd. Optical microscopy image of ≈750 nm HfO_2_ nanoparticle film grown on SiO_2_/Si substrate b) before and d) after CVD in CH_4_/H_2_ at 950 °C. HRTEM images of the HfO_2_ nanoparticles scratched from the film c) before and e–g) after CVD. Insets in (b) and (d) show the FFT analysis of the respective HfO_2_ nanoparticles. h) Raman spectra for the HfO_2_ nanoparticles before and after CVD with CH_4_ and H_2_ at 950 °C. Reproduced with permission.^32^ Copyright 2011, Wiley‐VCH.

The metal‐catalyst free direct CVD growth of graphene on ZrO_2_ substrates can be achieved at a temperature lower than 480 °C as reported by Scott et al.^31^ Growth was carried out on ZrO_2_ substrates using acetylene (C_2_H_2_) as a precursor. As soon as the substrate was placed, the reaction chamber was evacuated followed by the flow of Ar. The substrate was heated to a temperature of 325–650 °C. The flow of Ar was maintained for 10 min and then C_2_H_2_ was introduced into the chamber. X‐ray photoemission spectroscopy (XPS) confirmed the formation of zirconium oxide being present in the form of monoclinic baddeleyite, and being slightly oxygen deficient. The low temperature enables catalytically active oxides to form sp^2^ carbon. Wang et al.^27^ reported the metal‐catalyst free direct growth of vertically aligned graphene sheets (VGs) on ZrO_2_ substrate using either CH_4_ or ethanol (C_2_H_5_OH) as a precursor via thermal CVD. First, the substrate was mounted to the central region of the quartz tube, and heated from room temperature to 1130 °C in 50 min with 50 sccm H_2_ and 50 sccm Ar. Second, the substrate was annealed for 20 min at 1130 °C, and then a certain flow rate of CH_4_ or C_2_H_5_OH vapor was introduced into the chamber to initiate graphene growth. 2D or 3D growth of graphene could be controlled by altering feedstock concentration and reaction time (growth mechanism is described in Section 3.1.2). A typical scanning electron microscopy (SEM) image of VG sheets on ZrO_2_/Si is shown in **Figure** **3**a. Till now, direct growth of VGs is mainly achieved by PECVD techniques. Hence, it is observed that plasma plays a crucial role for vertical alignment of graphene sheets. Thus, this work paves a new avenue toward the development of a novel and reliable technique for direct sysnthesis of VGs, and therefore brings a plethora into the intrinsic mechanism of vertical graphene synthesis.

### 2.3 Graphene on HfO_2_ Substrates

The metal‐catalyst free direct growth of few layer graphene on HfO_2_ nanoparticles was achieved by using APCVD technique.^32^ The monoclinic HfO_2_ nanoparticles neither form metal nor carbide during nucleation in graphitic domains. The samples were heated in the presence of pure Ar at 900–950 °C and for 10 min in pure H_2_, and finally exposed to a CH_4_/H_2_ mixture for 20 min. Figure 3b,d shows optical microscopy images of the HfO_2_ nanoparticles film treated at 950 °C. The color of the deposited nanoparticles film was found to be white and it gradually became black after CVD growth, which indicated the deposition of carbon on the particles. Figure 3c shows high resolution transmission electron microscopy (HRTEM) images of the nanoparticles before CVD. The average particle size was found to be ≈4 nm. A fast Fourier transform (FFT) analysis (inset of Figure 3c) revealed the formation of monoclinic HfO_2_ nanoparticles. The HRTEM images of the nanoparticles after CVD technique are shown in Figure 3e–g.

The HfO_2_ nanoparticles were found to be encapsulated by 2–3 layers of graphitic carbon (Figure 3e). No phase transition occured in the HfO_2_ nanoparticles during CVD process. Particularly, no metallic hafnium or hafnium carbide was observed during post‐CVD FFT analysis. This suggested that the nanoparticles were of monoclinic HfO_2_ phase (inset of Figure 3c). The HfO_2_ nanoparticles were coated by multilayer graphitic carbon (Figure 3f). Two layers of graphitic carbon anchored along (111) direction of the HfO_2_ nanoparticles for a projection along (011) plane (Figure 3g). 1–5 layers of carbon showed an interlayer spacing of ≈0.35 nm corresponding to few‐layer nanographene (FLG). The HfO_2_ nanoparticles before CVD treatment showed no signatures of carbon in Raman study, whereas the post CVD samples exhibited G (≈1600 cm^−1^, full width at half maxima (FWHM) = 86 cm^−1^), D (≈1360 cm^−1^, FWHM = 83 cm^−1^), and 2D (≈2700 cm^−1^, FWHM = 88 cm^−1^) bands (Figure 3h). Nanosized graphitic domains on nanoparticles of a high‐k dielectric material find its limitations in terms of direct application for integration into electronics, however, they act as a model system for catalytic CVD of graphene on oxides. Hence, HfO_2_ is an interesting platform for basic studies pertaining to growth as well as future integration of graphene into the electronic devices.

### 2.4 Graphene on Hexagonal Boron Nitride Substrates

**Figure 4:**
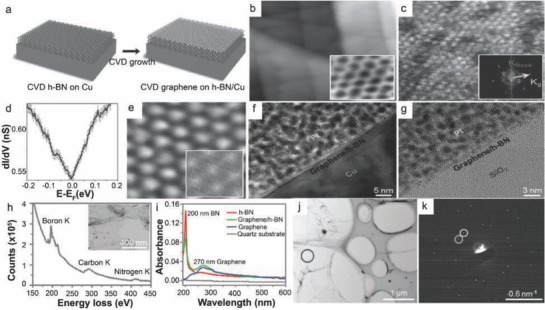
a) Schematic of the CVD grown graphene on h‐BN film/Cu foil. b) STM image with the straight step edges of the underlying h‐BN layers. Image area: 60 nm × 60 nm; parameters: *V*_b_ = 1.0 V, *I*_t_ = 0.1 nA. Inset: STM image of the graphene honeycomb lattice. c) STM image related to the Moiré pattern with a 0.55 nm period. Inset: The FFT result of (c). Image area: 10 nm × 10 nm; parameters: *V*_b_ = −0.8 V, *I*_t_ = 0.12 nA. d) Average value of the d*I*/d*V* data was determined on the region of 0.55 nm Moiré pattern with the error bars overlaid at each energy point; parameters: *V*_b_ = 0.2 V, *I*_t_ = 0.2 nA. e) STM image showing the Moiré pattern having period of 4.2 nm. Image area: 23 nm × 23 nm. Parameters: *V*_b_ = − 0.14 V, *I*_t_ = 0.11 nA. Inset: A simulated Moiré pattern (4.2 nm) having angle of 3.2° between the graphene and the h‐BN. Cross‐sectional TEM images of f) CVD‐grown graphene/h‐BN film on Cu foil; g) CVD grown graphene/h‐BN film on SiO_2_/Si substrate. h) EELS spectrum indicates the elements B, N, and C. Inset: Top view TEM image of graphene/h‐BN film. i) UV–Vis spectroscopy of h‐BN grown by CVD technique. j) TEM image of a graphene/h‐BN film. k) SAED pattern of graphene/h‐BN. Reproduced with permission.^42^ Copyright 2013, Wiley‐VCH.

Considerable research interest was gained for the h‐BN because its lattice parameter was found to be same with graphene.^33^, ^34^ When CVD grown graphene was transfered onto CVD‐grown h‐BN, a device on h‐BN having large area of graphene and threefold high mobility than that on SiO_2_ was fabricated.^35^ Graphene on h‐BN structures can be transferred either by mechanical exfoliation or via CVD growth onto h‐BN layers.^35^, ^36^, ^37^, ^38^ The metal‐catalyst free direct CVD growth of graphene onto h‐BN film was demonstrated in order to achieve pristine graphene/h‐BN interfaces with high area coverage. BN layer grown by CVD technique on Cu foil was taken as the substrate. The growth of graphene on h‐BN/Cu at 1000 °C for 40 min with 5 sccm H_2_ and 20 sccm CH_4_ at a total pressure of 210 mTorr was achieved. The structure of CVD graphene on h‐BN film/Cu foil is shown in **Figure** **4**a. It was found that h‐BN layers were coated with a single layer graphene (SLG) (Figure 4b). Honeycomb lattice of graphene was grown (inset Figure 4b) and owing to the same kind of atomic structures, a hexagonal Moiré pattern having a period of 0.55 nm was observed (Figure 4c). The stacking angle between graphene and h‐BN layers was observed to be 26° for 0.55 nm period.^39^ Electronic states of graphene were supported by the inert and flat h‐BN layers, similar to that of intrinsic graphene.^33^, ^40^, ^41^ d*I*/d*V* spectrum was determined by the scanning tunneling spectroscopy (STS) analysis which in turn was conducted through Moiré pattern region of 0.55 nm (Figure 4d). Spectrum having a sharp and symmetric V‐shape found its consistency to that of intrinsic graphene. Moiré pattern with a period of 4.2 nm and an angle of 3.2° between graphene and BN lattices was observed in the scanning tunneling microscopy (STM) image (Figure 4e).

Honeycomb lattice, Moiré patterns, sharp and symmetric V‐shape spectrum with the Dirac point at the Fermi level gave a clear notion that a high quality monolayer graphene was grown on h‐BN/Cu without any contribution of charge or doping. Cross‐sectional transmission electron microscopy (TEM) image confirmed the presence of a graphene/h‐BN film having a thickness of 2 nm (Figure 4f). The image dictated layered structure of graphene/h‐BN (Figure 4g). In the electron energy loss spectroscopy (EELS) spectrum (Figure 4h), three distinct edges of 200, 290, and 410 eV were observed, which indicated the characteristic K‐shell ionization edges of B, C, and N, respectively. Two distinct peaks at 200 and 270 nm appeared due to optical bandgap^43^ and π plasmon peak,^44^ respectively, and thus as‐grown graphene/h‐BN sample indicated the coexistence of graphene and h‐BN (Figure 4i). Figure 4k shows selected‐area electron diffraction (SAED) pattern of graphene/h‐BN in the marked zone (Figure 4j). The obtained data described two sets of hexagonal diffraction data in graphene/h‐BN sample, and hence confirmed the coexistence of single crystal graphene and h‐BN.

Similarly, Ding et al.^45^ investigated a few layer graphene on h‐BN substrate using CH_4_ as a precursor via metal catalyst free CVD technique. Stacked‐layers on h‐BN substrate using hexane (C_6_H_14_) (vapor) as a precursor via APCVD technique was demonstrated by Liu et al.^46^ However, it was observed that the sequential CVD is a robust technique for the direct growth and fabrication of stable graphene/h‐BN hybrid structure onto CVD grown h‐BN film on Cu. Also, it is possible to grow uniformly distributed, large‐scale SLG directly on h‐BN films. Moreoever, CVD grown graphene/h‐BN devices exhibit superior carrier mobility and reduced defects compared to mechanically transferred graphene onto h‐BN film. Furthermore, CVD‐grown graphene/h‐BN films were found to be significantly versatile to sustain during postgrowth transfer process. There is a flurry of demand for the development of high performance electronic devices using large area graphene/h‐BN hybrid structures having negligible defects.

### 2.5 Graphene on Quartz Substrates

**Figure 5:**
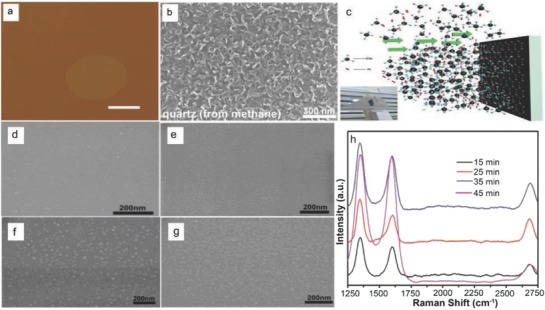
a) AFM images of graphene grains on quartz substrate. Scale bar 500 nm. Reproduced with permission.^28^ Copyright 2014, Wiley‐VCH. b) SEM images of VGs on quartz at a condition of 8 sccm CH_4_, 50 sccm H_2_, and 50 sccm Ar for 6 h. Reproduced with permission.^27^ Copyright 2017, Elsevier Ltd. c) Schematic illustration of graphene growth on glass substrate by vertical‐glass model (the inset shows the vertical glass substrate set up). High‐magnification SEM images of graphene islands on glass surface with growth time of d) 15 min, e) 25 min, f) 35 min, and g) 45 min. h) Raman spectroscopy of graphene grown on glasses with different growth time. Reproduced with permission.^47^ Copyright 2018, Elsevier Ltd.

Owing to the high melting point along with structural stability, quartz has a flat surface having roughness about 0.414 nm. Chen et al.^28^ demonstrated the metal‐catalyst free direct growth of single crystal hexagonal and dodecagonal pattern on quartz substrate using CH_4_ as a precursor via near equilibrium CVD technique. Quartz having smooth face toward the downward side was placed in a high‐temperature horizontal silica tube furnace. The furnace was heated at 1180 °C and stabilized for about 30 min in 250 sccm H_2_ and 300 sccm Ar. During entire growth, a gas mixture (CH_4_:H_2_ = 1.9–2.3:50) was taken as the carbon source. AFM phase image of graphene grain on quartz substrate is shown in **Figure** **5**a. Zhang et al.^24^ demonstrated the direct growth of uniform graphene films onto quartz substrate using CH_4_ as a precursor via remote (r)‐PECVD technique. Nanographene films having good uniformity were grown on 4 inch wafer. By controlling the growth duration, the value of transmittance on quartz was found to be greater than 92%, whereas it exhibited a low resistance of 40 kΩ sq^−1^ at 550 nm. The direct growth of VGs on quartz substrate can also be achieved using either CH_4_ or ethanol (C_2_H_5_OH) as a precursor via thermal CVD, as demonstrated by Wang et al.^27^ SEM image of VGs on quartz substrate is shown in Figure 5b (growth mechanism is described in Section 3.1.2).

Recently (2018), Chen et al. demonstrated direct growth of graphene on vertically placed quartz substrates via APCVD.^47^ Figure 5c is the schematic of the vertical‐quartz substrate (20 mm × 20 mm × 1 mm) APCVD model. The substrate was heated to 1000 °C and stabilized for about 10 min under Ar (100 sccm) and H_2_ (20 sccm) and then CH_4_ (10 sccm) was introduced for 15, 25, 35, and 45 min, respectively. Figure 5d–g shows the SEM images of the graphene islands on quartz substrates grown for 15, 25, 35, and 45 min, respectively. Large number of graphene nanocrystals (<10 nm) were observed on the quartz surface. As the growth time increased from 15 to 35 min, the size of the graphene islands increased (10–30 nm). Finally, quartz surface was mainly covered by the larger graphene islands as the growth time increased to 45 min (Figure 5g). The density of the mixed gas facing the quartz substrate was greater as compared to the surrounding gas which significantly enhanced the collision probability of the reactive fragments with the quartz surface, and finally led to the higher growth rate of graphene on the front surface. For all the samples, the intensity of the D peaks was higher as compared to the G peaks as observed from Raman spectra (Figure 5h), and the intensity ratio of the G peak to the 2D peak was >1. It was inferred that a large number of defects were present and the grown graphene was multilayered. These findings indicated that these materials have potential applications in future transparent and conductive electronics. The direct growth of large size and high‐quality graphene on quartz substrates with a clean, wrinkle‐free, and breakage‐free morphology is important for fundamental research and practical applications.

### 2.6 Graphene on Si_3_N_4_ Substrates

**Figure 6:**
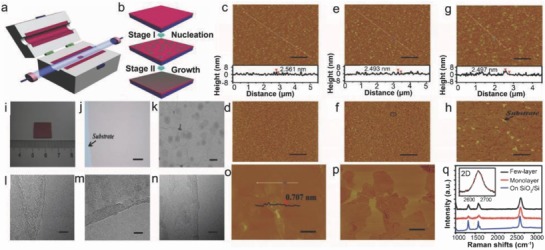
a) Schematic diagram of the CVD system where graphene growth on a Si_3_N_4_ substrate was carried out. b) Schematic representation of the two‐stage process for graphene growth. c,d) AFM images of Si_3_N_4_ substrate. e,f) AFM images of the Si_3_N_4_ substrate after nucleation. g,h) AFM images of the Si_3_N_4_ substrate. c,e,g) Height images and d,f,h) phase images are shown. Scale bars = 1 µm. i) Photograph of graphene film on Si_3_N_4_ substrate. j) Optical image of a graphene film on a Si_3_N_4_ substrate. Scale bar 50 µm. k) SEM image of graphene film. Scale bar = 500 nm. l–n) HRTEM images of graphene films. Scale bars: 5 nm. o,p) AFM images of transferred graphene on SiO_2_/Si substrates: height image (o), phase image (p); scale bars = 300 nm. q) Raman spectra of the graphene films on a Si_3_N_4_ substrate and the transferred graphene on a SiO_2_/Si substrate. The inset shows the enlarged 2D peak of single‐layer graphene. Reproduced with permission.^48^ Copyright 2013, Wiley‐VCH.

The metal‐catalyst free direct growth of large‐area graphene films on silicon nitride (Si_3_N_4_) substrates can be achieved by a two‐stage CVD process.^48^ Graphene in the form of sheets can successively grow on Si_3_N_4_ surface, and at a later stage merged together to form a polycrystalline graphene film. These graphene films can be used to fabricate field‐effect transistors (FETs). A clean Si_3_N_4_/SiO_2_/Si substrate was kept into a quartz furnace at high‐temperature (**Figure** **6**a). The growth was followed by the nucleation of graphene and subsequently the growth of graphene on a Si_3_N_4_ surface (Figure 6b). The flow rates of CH_4_ and Ar were taken as 2.3 and 300 sccm, respectively. The formation of discrete graphene nanocrystal on the Si_3_N_4_ substrates was observed. CH_4_ and H_2_ in the ratio of 5:50 were used as the carbon source for the growth of high‐quality graphene film. Figure 6c–h depicts the AFM images of Si_3_N_4_ surface profile before and after graphene growth. The polycrystalline Si_3_N_4_ layer was deposited on SiO_2_/Si substrate via low pressure CVD (LPCVD) (Figure 6c,d). The average roughness of the Si_3_N_4_ surface was found to be 0.702 nm, whereas height of raised Si_3_N_4_ particles was about 2.561 nm (Figure 6c). The variation was found in the surface roughness (*R*_a_ ≈ 0.775 nm) (Figure 6e). The surface underwent rigorous modifications and was coated with graphene nanocrystals (Figure 6f). The AFM image (Figure 6f) is marked with black circle, which indicated contrast between the graphene and the Si_3_N_4_ substrate. The lateral dimensions of the graphene nanocrystals were observed to be of 30–40 nm. The morphology of graphene is shown in AFM image (Figure 6g).

The film was not distinguished properly from the underlying Si_3_N_4_ layer caused by the surface roughness (*R*_a_ ≈ 0.887 nm). The graphene film was observed to be clearly separated from Si_3_N_4_ substrate, and formation of wrinkles took place along the boundary of the sheets (Figure 6h). It was confirmed that polycrystalline substrate did not affect the growth of graphene. It was observed that graphene covered the surface at the end of CVD growth (Figure 6i). The graphene films were uniformly distributed as shown in Figure 6j. SEM image of the graphene film is shown in Figure 6k. A clear layered structure was observed in the TEM images (Figure 6l–n). Figure 6o,p shows AFM images of transferred graphene on SiO_2_/Si substrates. The line scan (Figure 6o) illustrates that the step height between the surface of the sheet and the substrate was about 0.707 nm. Raman analyses also (Figure 6q) confirmed the growth of single and few‐layer graphene. The carrier mobility (1510 cm^2^ V^−1^ s^−1^ in air, 1518 cm^2^ V^−1^ s^−1^ in N_2_) was found to be three times higher than those grown on SiO_2_/Si substrates as well as better than graphene grown using metal catalyst.^23^, ^49^ Thus, large area high quality graphene films were grown on Si_3_N_4_ substrates via two‐stage metal catalyst‐free CVD process where the detrimental effects of the substrate were minimized. In addition, the graphene sheets were successively grown along rough Si_3_N_4_ surface followed by merging together to form a high quality polycrystalline graphene film. The difficulties that arise during postgrowth transfer method can be eleminated by adopting two‐stage metal‐catalyst‐free‐growth technique, which finds its compatibility with current Si processing technology.

### 2.7 Graphene on AlN Substrates

Aluminum nitride (AlN) is a promising material due to its versatile applications such as microelectronic and optoelectronic devices, short wavelength emitters, electronic packaging, and acoustic wave resonators.^50^ However, a semiconductor template is pertinent in order to grow high quality graphene. In this regard, AlN grown on Si finds its potential as a suitable template by replacing 3C‐SiC for wide range of nitride and UV application. Direct growth of graphene on AlN/Si template was carried out by Michon et al.^51^ In this study, graphene was grown on AlN/Si (111) templates via propane CVD, where N_2_/H_2_ mixture was used as the carrier gas. A rotational disorder along with wrinkles was formed onto the graphene films grown on AlN/Si. Here, temperature played an important role to improve the structural quality of the film. Again, high temperature growth might have influenced rough surface, but there was no impact on the structural quality of the graphene film as determined by Raman analysis. Moreover, the temperature for optimum growth might be higher (1350 °C), which suggests the growth of high‐quality graphene on bulk AlN substrates. Furthermore, during processing, AlN etching was minimized by the growth of graphene compared to the annealing (i.e., without propane), which enabled the growth of AlN at 1250 °C for 6 min without etching effects. It may be mentioned here that thermal treatment of nitrides films finds an ease with this method to enhance the quality of crystal, and favors the activation of doping at the time of ion implantation.^51^ Thus, growth of graphene on semiconductor template without using carbon based derivatives opens up a new possibility of direct growth.

### 2.8 Graphene on MgO Substrates

**Figure 7:**
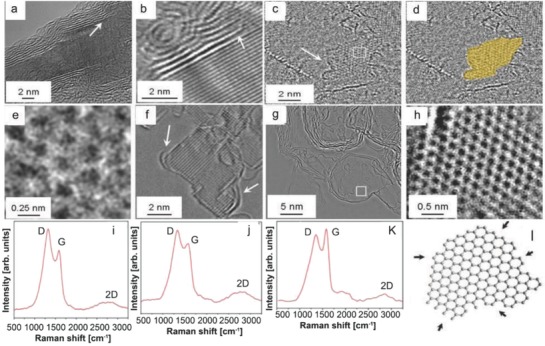
a,b) TEM images of few‐layer graphene on MgO crystal. c,d) Formation of graphene island on the surface of a MgO crystal. e) Magnified region from the box in panel (c) indicating the graphene structure. f) Cross‐sectional image of graphene on the surface of an MgO crystal. g) Growth of graphene shells when MgO is removed. h) Magnified region from the box in panel (g) highlighting graphene structure. Raman spectra of i,j) nanographite and k) a purified sample prepared at 325 °C over MgO. l) Schematic representation of a nanographene flake. Reproduced with permission.^53^ Copyright 2010, American Chemical Society.

MgO is considered to be advantageous for the growth of nanographene and few‐layer nanographene (nFLG) directly using CVD technique. However, calibration of the reaction time or temperature needs to be undertaken to grow nFLG and nanographene. The growth can be done at temperatures of 325 °C using acetylene as a precursor.^31^ **Figure** **7**a–h shows TEM images of the samples prepared by catalytic CVD reactions on MgO using cyclohexane as the feedstock. Figure 7a,b shows the formation of MgO crystal at 875 °C after CVD treatment where reaction was undertaken for 5 min. The interspacing between graphitic layers (2–10) was found to be 3.5 Å. Alignment of the graphitic layers with MgO lattice planes^100^ had a spacing of 0.21 nm. It was also observed that the graphene layers were attached to the MgO crystal. This observation was found to be same as the graphitic layers growth by SiC decomposition.^52^ The number of graphene layers formed was 2 and 10 at reaction times of 5 min and 1 h, respectively. Growth of graphene nanoislands on the surface of oxide crystals and nanographene shells is shown in Figure 7c–f and Figure 7g,h, respectively. A strong D mode and broadened G mode along with a weak and broad 2D mode were observed in the Raman spectra (Figure 7i–k). A large number of edge states existed in nanographene compared to its bulk counterpart (Figure 7l).

Ferrari^54^ investigated the effects of edge states in detail, which were mainly defects. These effects exactly matched with Raman spectra along with nanographitic species. Gaddam et al.^55^ reported monolayer on MgO substrate using C_2_H_4_ as a precursor via free radical assisted CVD. Zhao et al.^56^ reported single or few layers on MgO substrate using benzene and pyridine as a precursor via APCVD. Thus, MgO is found to be a suitable candidate for the metal‐catalyst free direct growth of nanographene through CVD technique. Moreover, the growth can be undertaken at low temperature (325 °C) using this technique. Owing to low temperature growth, this technique is promising for the growth of large area nanoribbon graphene using present Si‐based technologies.

### 2.9 Graphene on SrTiO_3_ Substrates

**Figure 8:**
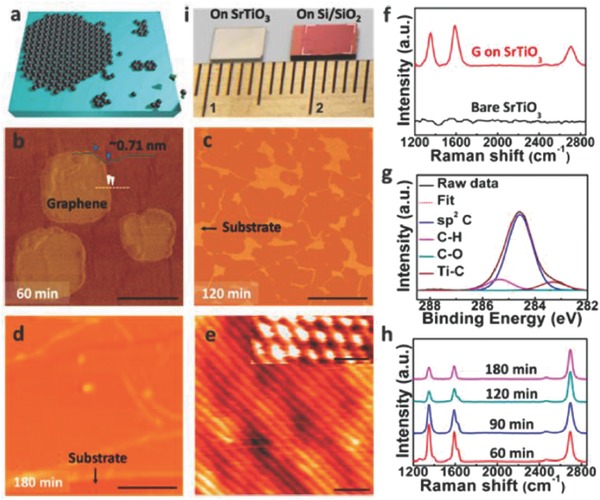
a) Schematic of graphene growth on STO (001) substrates. b–d) AFM images of the graphene grown by CVD technique for different duration. Scale bars: (b) 250 nm. (c,d) 1 µm. e) STM image (*V*_s_ = 0.7 µV, *I*_t_ = 18 nA) of a graphene film. Scale bar: 1 nm. Inset: STM image of graphene honeycomb lattice. Scale bar: 0.5 nm. f) Raman analysis of as‐grown graphene and bare STO substrates. g) XPS spectrum of as‐grown graphene. h) Raman analysis of graphene for the evolution of graphene growth. i) Photograph of an as‐grown graphene film on STO and a transferred graphene film to Si/SiO_2_ substrate. Reproduced with permission.^63^ Copyright 2014, American Chemical Society.

The use of high‐k dielectric as a substrate by replacing low‐k (SiO_2_) leads to better gate modulation, improved gate capacitance, and reduced gate leakage.^57^ However, the difficulties associated with SiO_2_ are carrier scattering due to charge fluctuations and surface roughness. These results are obstruction for further integration of FETs based on SiO_2_‐gated graphene.^57^, ^58^, ^59^ Therefore, direct growth of graphene on the surface of high‐k dielectric and scatter‐screening dielectric substrates becomes significant. SrTiO_3_ (STO), a transparent, high‐k perovskite dielectric may have great thermal stabilities and potentials.^60^, ^61^, ^62^ Sun et al. demonstrated the metal‐catalyst free direct CVD growth of graphene on STO (001) substrates using a simple APCVD technique (Ar/H_2_/CH_4_:100/50/2.5 sccm).^63^ They successfully fabricated STO‐gated bipolar FET and studied their low voltage operation along with magnetotransport properties of as‐grown graphene/STO samples. The growth of graphene on STO substrate was attributed to the in‐plane propagation process of carbon species (**Figure** **8**a). AFM images (Figure 8b–d), taken from the graphene nanoislands, exhibited the formation of continuous film with irregular voids along with complete layers of graphene wrinkles obtained for 60, 120, and 180 min, respectively. The height of the nanoislands was found to be ≈0.71 nm, and the formation of a uniform film may be attributed to the size elongation of nanoislands followed by the agglomeration. From STM images of the as‐grown graphene on STO (Figure 8e and inset), the lattice constant was determined to be ≈0.246 nm. Three distinct peaks were observed in the Raman spectrum of as‐grown graphene on STO (Figure 8f). The peaks corresponding to D, G, and 2D bands were located at 1350, 1597, and 2695 cm^−1^, respectively. However, the 2D peak of graphene grown on the STO substrate did not appear due to the substrate screening effect (lattice mismatch and/or strong interaction due to the chemical bonding between the graphene and substrate). XPS analysis (Figure 8g) confirmed the signature of Ti–C peak, sp^2^ carbon peak, and C–H peak at 283.4, 284.8, 285.3 eV, respectively.

High quality and single layer graphene was grown after CVD treatment for 180 min (Figure 8h). Photograph of the as‐grown and as‐transferred samples on Si/SiO_2_ (Figure 8i) indicated that STO substrate size limits the area of entire monolayer having uniform contrast. Recently, Karamat et al.^64^ and He et al.^65^ reported the growth of nanographene and few‐layer graphene shell on SrTiO_3_ using CH_4_ as a precursor via APCVD method, respectively. Thus, from the above discussion it is evident that large‐area monolayer graphene with good uniformity can be grown on single crystal STO substrates directly using CVD techniques. Owing to unique electronic and optical properties of the as‐grown graphene on STO, it holds a strong potential toward energy‐saving devices, high‐performance FETs, and transparent electrodes. Therefore, from device point of view, further studies need to be undertaken in this area especially its growth optimization, band structure, and chemical bonding with the substrate.

### 2.10 Graphene on Al_2_O_3_ Substrates

**Figure 9:**
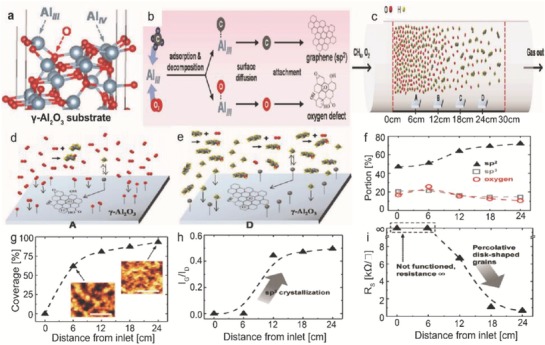
a) Structure of the γ‐Al_2_O_3_ surface. b) Schematic flow process of CH_4_ or O_2_ to graphene or oxygen defect through adsorption on Al III sites. c) Change of gas composition within a CVD heating zone. Gas composition at a location d) closer and e) further from the oxygen influx. f) Comparison of sp^2^, sp^3^, and oxygen portion of graphene with respect to the distance from the inlet. g) Coverage variation of graphene grown on γ‐Al_2_O_3_ with respect to the distance from the inlet. Plots of h) *I*_G_/*I*_D_, and i) *R*_S_ for the graphene with respect to the distance from the inlet. Reproduced with permission.^82^ Copyright 2017, Wiley‐VCH.

Generally, CVD growth of graphene involves metal catalyst such as Cu or Ni^66^, ^67^, ^68^, ^69^, ^70^ and there exists strong adhesion between the metal and the graphene which causes several issues such as contaminations in the product, undesired doping by the metal ions, and chemical exposure in the metal etching, and polymer residues from the transfer process.^71^, ^72^, ^73^ Ceramic catalyst^25^, ^31^, ^53^, ^74^ was proposed as a metal‐free alternative. One of the most promising candidates is γ‐Al_2_O_3_ due to its insulating properties, lower adhesion energy to graphene, and reusability as a catalyst.^75^, ^76^ A dielectric ceramic material such as γ‐Al_2_O_3_ can itself serve as a device substrate, where a device may be directly fabricated onto the synthesized graphene without any need for a transfer process. The γ‐Al_2_O_3_ has highly reactive tri‐coordinated Al (Al‐III) sites on the surface (**Figure** **9**a), which acts as catalytic sites due to strong reactivity by adsorbing various molecules.^77^, ^78^ During an actual graphene synthesis, CH_4_ was used as the carbon precursor, whereas O_2_ from the leakage reacted to the Al‐III sites of γ‐Al_2_O_3_. These were decomposed to generate adatoms, which attached themselves for graphene nucleation through surface diffusion.^76^, ^78^, ^79^ The carbon adatoms soon progressed into sp^2^ crystallization; the oxygen adatoms instead developed oxygen defects and consequently acted as a growth inhibitor (Figure 9b). Figure 9c shows the gradient change of the gas composition with respect to the substrate positions within a CVD heating zone. When the gas molecules passed through the actual heating zone of a tube furnace in the CVD system, heat was generated through spontaneous dehydrogenation followed by the polymerization and elongation.^80^ More C*_x_*H*_y_*‐type linear hydrocarbons were produced while travelling along the gas flow, which then reacted with O_2_ in the following manner^81^

(1) $$2{\mathrm{CH}}_{4}+O\to C_{2}\mathrm{HO}+3H_{2}+H$$

(2) $$C_{2}H_{2}+{\mathrm{CH}}_{4}+O_{2}\to C_{2}\mathrm{HO}+{\mathrm{CH}}_{2}O+3H$$

(3) $$2C_{2}H_{2}+O\to C_{3}H_{2}+H_{2}+\mathrm{CO}$$

These reactions created a difference in O_2_ concentration along the heating zone (Figure 9d,e). As the substrate was placed further from the inlet, the source of the O_2_ leakage, the oxygen, and sp^3^ portions showed a decreasing trend, ranging from 25.76% to 10.59%, and from 21.15% to 14.70%, respectively (Figure 9f). The graphene formation was strongly suppressed near to the inlet or when exposure to O_2_ was at the highest (Figure 9g). The grown graphene was composed of the nanosized grains connected to one another. The values of *I*_G_/*I*_D_ corresponding to the sp^2^ portion/defect with respect to the substrate position from inlet are shown in Figure 9h. The sp^2^ crystallization was significantly enhanced at distances over 6 cm, which was consistent with the XPS results (Figure 9f). The samples grown at 0 and 6 cm position had the sheet resistance (*R*_S_) values at infinity due to the insufficient graphene growth (Figure 9i), which was apparent from the low percolative graphene coverage of 0% and 61.7%, respectively (Figure 9g). The sample grown at 6 cm position formed small disk‐shaped graphene grains without any overlap to form an electrical percolative channel. The samples grown at the position of 12, 18, and 24 cm exhibited the *R*_S_ values of 6.64, 1.06, and 0.64 kΩ sq^−1^, respectively. From the above discussion, it was oberserved that a quantitative and systematic analysis helps to clearly elucidate the impact of oxygen exposure on the growth as well as device characteristics of graphene grown on γ‐Al_2_O_3_ using CVD technique.

The graphene is considered to play a pivotal role in optoelectronic devices as a transparent conducting film (TCF). However, the manufacturing process available in the literature utilizes graphene as a TCF which follows transfer procedures. These steps are time‐consuming and sometimes chemical contaminations are encountered during processing which may impose detrimental effects to optoelectronic devices. Therefore, the metal‐catalyst free direct CVD growth of graphene on crystalline Al_2_O_3_ (sapphire) was demonstrated by many researchers. Chen et al.^28^ reported single crystal hexagonal and dodecagonal patterns on sapphire substrate using CH_4_ as a precursor via near equilibrium CVD technique, whereas Song et al.^83^ reported the growth of single layer graphene on sapphire substrate using CH_4_ as a precursor via metal catalyst‐free APCVD. Zhang et al.^24^ studied uniform graphene films on sapphire substrate using CH_4_ as a precursor by PECVD technique. A study pertaining to graphene crystal onto sapphire substrate was accomplished using C_2_H_4_ as a precursor using PECVD technique.^26^

### 2.11 Graphene on Glass and Mica Substrates

The metal‐catalyst free direct growth of graphene on glass and mica substrates can be achieved via r‐PECVD system at a substrate temperature of ≈550 °C using pure CH_4_ as a precursor.^24^ The growth temperature (550 °C) was found to be lower than the temperatures for CVD graphene growth (≈900–1000 °C).^84^, ^85^, ^86^, ^87^ Therefore, it enabled the growth of graphene on glass substrates. Moreover, direct growth of nanographene films can be done on any substrate using this technique. The growth of graphene films on atomic layer deposited mica and 4 inch glass wafers was also demonstrated. The growth rate on different substrates changed to some extent due to different adsorption rate, as plasma can dissociate CH_4_ into different species such as CH*_x_*, C_2_H*_y_*, C_3_H*_z_*, and atomic hydrogen. These radicals could play a pivotal role during nucleation followed by the growth of nanographene. Apart from the above discussions in Sections 2.5, 2.10, and 2.11 about the direct CVD growth of graphene on transparent substrates, reports are also available in the literature where uniform graphene films were grown on glass substrates using PECVD technique.^88^ High temperature CVD growth of nanographene on quartz (transparent substrate) was carried out without using any metal catalyst.^89^ Furthermore, high‐temperature direct CVD growth of graphene on quartz and sapphire substrates was undertaken and future prospects of the transfer‐free graphene for transparent electrodes were explored.^90^

### 2.12 Graphene on TiO_2_ Substrates

**Figure 10:**
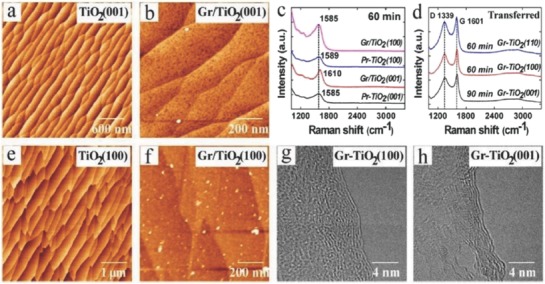
Graphene is grown by CVD technique on r‐TiO_2_ (100) and (001) substrates. a,e) AFM images of the pristine TiO_2_ after cleaning. b,f) AFM images of the graphene‐TiO_2_ film grown by CVD technique for 30 min. g,h) TEM images are taken from the graphene films stripped on TiO_2_ (Gr‐TiO_2_). c) Raman spectra of the graphene films taken from different TiO_2_ substrates. d) Raman spectra of the graphene films deposited on SiO_2_/Si substrates. Reproduced with permission.^93^ Copyright 2016, American Chemical Society.

Recently, photocatalysis effect based on titania (TiO_2_) has gained tremendous research interest due to its green impact on environment.^91^, ^92^ Direct fabrication of graphene on TiO_2_ surface results in the contamination free graphene‐TiO_2_ interface. Liu et al.^93^ demonstrated the metal‐catalyst free direct CVD growth of graphene films on the as‐prepared r‐TiO_2_ surfaces such as (001) and (100) faces. Both substrates were cleaned using wet chemical etching followed by flattening into atomic smoothness.^94^ The AFM images of the as‐prepared pristine surfaces are shown in **Figure** **10**a,e. After the CVD growth, both surfaces exhibited changes similar to that of the (110) substrate (small islands having low surface roughness) (Figure 10b,f). Raman spectra exhibited the features similar to carbonaceous species (Figure 10c). When graphene was transferred onto the SiO_2_/Si substrate, the sharp G‐bands and D‐bands of the graphene became prominent (Figure 10d). Graphitic layers were seen at the edges of the films (Figure 10g,h). Quality of the graphene structures grown on the (100) and (001) r‐TiO_2_ surfaces was found to be lower than the (110) plane for the same growth parameter. Similarly, Bansal et al.^95^ demonstrated direct growth of few‐to‐monolayer of graphene on TiO_2_ substrate using CH_4_ as a precursor via APCVD/LPCVD technique. Moreover, graphitic nanostructures were grown on titania nanowire aerogel at 750 °C via CVD using ethylene. Three layer thick graphitic nanostructures gave a clear indication that titania nanowire surface enabled the graphitization to few‐layer graphene. The growth mechanism of few‐layer graphene on nanostructured metal oxides paved a way toward facile and controllable processing of metal oxide‐nanocarbon fiber–shell composites.^96^ Since the heterostructure (graphene‐on‐titania) has applications in energy and electrochemical technologies, it is highly recommended to study its properties and performance in these applications as future work. The summary of the growth of graphene on different dielectric substrates is mentioned in **Table** **1**.

**Table 1:** Summary of different parameters for the direct growth of graphene on various dielectric substrates

Substrates	Deposition techniques	Precursors used	Type of growth	Flow rate [sccm]	Growth time [min]	Temperature [°C]	Morphology	Ref.
SrTiO_3_	APCVD	CH_4_	Direct	CH_4_ = 5, 3, 1.5, 0.5, H_2_ = 20, Ar = 100	20	1050	Few‐layered graphene shell	65
SrTiO_3_	Catalyst‐ free CVD	CH_4_	Direct	Ar:H_2_:CH_4_ = 100:50:2.5	60, 120 and 180	–	Uniform monolayer	63
SrTiO_3_	APCVD	CH_4_	Direct	CH_4_:Ar:H_2_ = 8:100: 50	180, 240, and 420	1000	Nanographene	64
h‐BN	Metal‐ catalyst‐free CVD	CH_4_	Direct	CH_4_:Ar = 50:180 to 90:180	3–8	1000	Few layers	45
h‐BN	APCVD	C_6_H_14_ (vapor)	Direct	–	10	950	Stacked layers	46
MgO	Free radical‐assisted CVD	C_2_H_4_	Direct	–	–	–	Monolayer	55
MgO	APCVD	Benzene, pyridine	Direct	Ar = 40	20	650, 700, 750, and 800	Single or few layers	56
MgO	APCVD (thermal CVD)	Acetylene	Direct	–	–	325–650	Single and multilayered nanoflakes	31
ZrO_2_	Thermal CVD	CH_4_	Direct	CH_4_:H_2_:Ar = 8:50:50	360	–	Vertically aligned graphene nanosheets (VAGNs)	27
ZrO_2_	APCVD (thermal CVD)	Acetylene	Direct	–	–	325–650	Single and multilayered nanoflakes	31
HfO_2_	APCVD	CH_4_	Direct	–	20	950	Few layers	32
Si_3_N_4_	Metal‐catalyst‐ free CVD	CH_4_	Direct	CH_4_:Ar:H_2_ = 2.3:300:5	60	1150	Polycrystalline films	48
Al_2_O_3_	PECVD	CH_4_	Direct	–	180	525	Uniform films	24
Al_2_O_3_	APCVD	CH_4_	Direct	–	–	–	Nanosized grains	82
Sapphire	PECVD	CH_4_	Direct	–	240	500	Uniform films	24
Sapphire	Metal‐ catalyst‐free APCVD	CH_4_	Direct	H_2_:CH_4_ = 50:30	120	950	Single layer	83
TiO_2_	APCVD/ LPCVD	CH_4_	Direct	H_2_ = 20, Ar = 300, CH_4_/Ar = 300	60	1100	Few‐to‐monolayer	95
TiO_2_	CVD	C_2_H_4_	Direct	–	–	–	Few layered graphene	96
Quartz	PECVD	CH_4_	Direct	–	300	500	Uniform films	24
SiO_2_, Si, and quartz	APCVD (thermal CVD)	CH_4_	Direct	CH_4_:H_2_:Ar = 8:50:50	60, 120, and 360	1130	VAGNs	27
SiO_2_	APCVD	CH_4_	Direct	CH_4_:H_2_:Ar = 3–10:10–50:450	60–120	1100–1200	Few‐layered Films	29
SiO_2_	PECVD	CH_4_	Direct	Ar/H_2_:CH_4_ = 20:3	30–120	400–700	Nanowalls	30
SiO_2_/Si and sapphire	PECVD	CH_4_ or C_2_H_4_	Direct	–	–	550–650	Crystal	26
SiO_2_/Si, Si_3_N_4_/SiO_2_/Si, quartz, ST‐cut quartz, and sapphire	Near equilibrium CVD	CH_4_	Direct	CH_4_:H_2_ = 1.9/2.3:50 Ar = 300	30	1180	Single crystal hexagonal and dodecagonal pattern	28
Glass	PECVD	CH_4_	Direct	Ar/H_2_:CH_4_ = 20:3	30–120	400–700	Nanowalls	30
Glass	PECVD	CH_4_	Direct	CH_4_ = 5.6, 6, 10	60	400–600	Uniform Films	88
Mica	PECVD	CH_4_	Direct	–	240	525	Uniform films	24

## 3 Catalyst‐Free Direct CVD Growth of Graphene on Technologically Important Semiconducting Substrates

Apart from Ge, there are only few reports available on the metal‐catalyst free direct CVD growth of graphene on important semiconducting substrates such as Si, GaN, and SiC. To the best of our knowledge, there are no reports avalaible on the metal‐catalyst free direct CVD growth of graphene on other important arsenide and phosphide based semiconductors. Therefore, in the successive sections, direct CVD growth mechanism and morphology of graphene on Si, Ge, GaN, and SiC substrates will be discussed. Detailed discussion is being presented for Si and Ge, as they are important semiconductors for the next generation graphene/Si and graphene/Ge based hybrid electronic devices.

### 3.1 Catalyst‐Free Direct CVD Growth of Graphene on Si Substrates

#### 3.1.1 Low Temperature Growth

**Figure 11:**
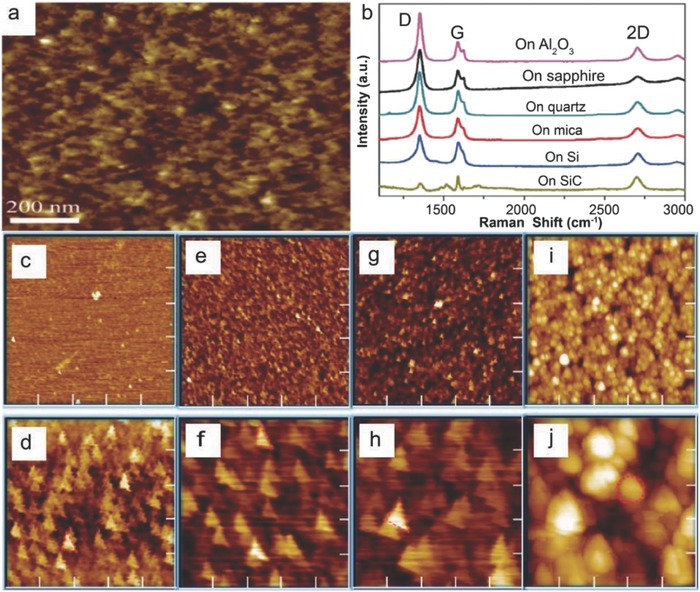
a) AFM image of nanographene film grown on Si at 525 °C, 0.204 Torr, for 3 h and b) Raman spectra of nanographene films grown on various substrates under different conditions. The plasma power was 100 W in each case. Reproduced with permission.^24^ Copyright 2011, Springer Nature. AFM images of nanographene directly grown on silicon substrates for c,d) 1 h at 800 °C, e,f) at 900 °C, g,h) 1000 °C, and i,j) 1100 °C using the topography mode except (d) (phase mode). The scan size is 5 µm × 5 µm for (c), (e), (g), and (i) and 1 µm × 1 µm for (d), (f), (h), and (j). Reproduced with permission.^89^ Copyright 2011, American Chemical Society.

Takami et al. reported the catalyst‐free direct growth of networked graphite on Si and SiO_2_ substrates by using photoemission‐assisted plasma enhanced CVD system.^97^ They grew MLG particles (diameter of ≈10 nm) on Si (001) substrates at 700 °C by using Ar‐diluted CH_4_. Particles were closely connected to each other, and shared some graphene sheets between them. The advantage of using this system is that the DC discharge plasma assisted by photoelectrons emitted from the substrate under ultraviolet (UV) light irradiation could be generated close to the substrate with a controllable volume. Similarly, Zhang et al. reported the catalyst‐free direct graphene growth on various substrates including Si and SiC.^24^ Nanocrystalline graphene was directly grown on Si substrate via *r*‐PECVD system at a relatively low temperature of ≈550 °C by using pure CH_4_. This graphene growth process was quite unconventional as compared to high temperature (≈900–1000 °C) CVD graphene growth.^85^, ^86^, ^87^ It also enabled direct deposition of graphene films on low melting point substrates. Nanographene growth on Si substrate was carried out at 525 °C, 0.204 Torr (CH_4_), for 3 h under the plasma power of 100 W. **Figure** **11**a is the AFM micrograph showing the morphology of directly grown graphene. Islands type nanographene growth was observed instead of uniform film. Raman spectra of the graphene on different substrates are shown in Figure 11b, which did not vary significantly for different substrates. It was observed that the graphene growth on SiC was much faster, and the crystal size was much bigger with low surface roughness and less lattice mismatch. This may be attributed to the fact that adsorption abilities of hydrocarbon radical species on different substrates in plasma are different, which results in the different growth rate on different substrates. In plasma, CH_4_ can dissociate itself into various reactive radicals, such as CH*_x_*, C_2_H*_y_*, C_3_H*_z_*, and atomic H_2_. These species play a key role during the nanographene nucleation and growth stages. The carbon‐containing radicals continuously get adsorbed onto the substrate surface, and bonded with each other by diffusion and collision to form graphene nanoclusters with H‐terminations. Nucleation on the as‐grown graphene was much easier as compared to clean substrates. Moreover, due to atomic H_2_, strong etching effect was there which helped in the suppression of formation of amorphous carbon. Similarly, some other low temperature (500 and 780 °C) direct CVD growth of multilayer graphene films and graphene nanowalls on Si substrates with the help of microwave surface wave plasma CVD and PECVD were reported by Adhikari et al. and Zhou et al., respectively.^98^, ^99^

#### 3.1.2 High Temperature Growth on Flat Substrates

Kim et al.^89^ reported the direct growth of graphene on 500 µm thick Si (100) wafers at high temperatures (800–1100 °C) by using thermal LPCVD system. Once the system pressure reached to 1 mTorr at the desired growth temperature, C_2_H_2_ and Ar with flow rates of 25 and 50 sccm, respectively, were introduced. Graphene growth at different temperatures was carried out by maintaining the pressure in the range of 2–100 Torr for 1 h. Figure 11c–h shows the AFM images of graphene grown at 800–1000 °C. Nanoscale triangle‐shaped planar graphitic carbon structures and triangle nanographenes (TNGs) were observed instead of a continuous uniform layer, whereas spherical nonplanar carbon clusters were observed at 1100 °C (Figure 11i,j). The right triangular shape of grown NG was anisotropic, and indicated that TNGs were crystalline in nature, as the crystal growth largely depended on the orientation owing to orientation‐dependent formation energy. TNGs became larger, as the growth temperature increased from 800 to 1000 °C (Figure 11c–h). They were like isosceles right TNG on Si, and the number density of the triangles was continuously reduced, which indicated that isosceles triangle formation on Si was more favorable, as the growth temperature increased. Thus, it was concluded that at high growth temperatures, the growth was less dependent on the diffusion than on the crystal orientation. However, spherical carbon clusters growth on Si was more favorable than the planar structure at 1100 °C (Figure 11i,j). This was due to the high thermal stress driven by the large thermal expansion coefficient with a significant lattice mismatch between graphene and Si.^100^, ^101^ The thermal stress relaxed by forming spherical graphitic clusters, which ultimately reduced the defect formation unlike the case for the planar structure. The position of the G bands in the Raman spectra of these TNGs grown at different temperatures was located at 1600 cm^−1^. This was higher than the typical large scale graphene or graphite (1580 cm^−1^), which confirmed the growth of NG.^102^

**Figure 12:**
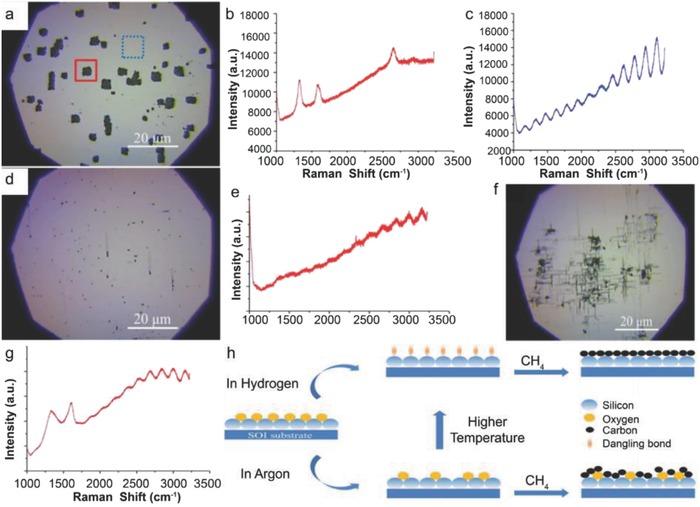
Graphene growth on SOI at 920 °C in hydrogen atmosphere. a) The optical image. The rectangle black dots (highlighted in solid red box) are covered with graphene, while the white area (highlighted in dashed blue box) is the unreactive SOI surface. b,c) Raman spectra collected in the solid red and dashed blue box, respectively. The three peaks located at 1331, 1589, and 2650 cm^−1^ are, respectively, associated with the D, G, and G′ modes of graphene. The oscillation curve in (c) is due to optical interference in the sandwich structure of SOI. The peaks at 1000 cm^−1^ are from silicon. d,f) The optical images of SOI after graphene growth at 870 and 895 °C, respectively. e,g) The Raman spectra detected at the black spot in (d) and (f). h) Schematic of graphene growth mechanismon SOI substrate. Reproduced with permission.^15^ Copyright 2012, American Institute of Physics.

Moreover, Hong et al. attempted the metal‐catalyst free direct growth of graphene on Si substrates, eventhough they had succeeded in growing few‐layers graphene films on Si‐on‐insulator surface (SOI), but they were in the form of small dots and patches instead of continuous uniform graphene films.^15^ A number of growth experiments were carried out in a LPCVD system. The SOI substrates were heated in H_2_ atmosphere with a flow rate of 6 sccm at 300 mTorr and then kept for 20 min to activate the substrate surface, followed by 35 sccm of CH_4_ for 30 min at desired growth temperatures (870–970 °C). After growth at 920 °C, two kinds of surface features were observed: 1) the rectangular black dots highlighted by the solid red box, and 2) the large white area highlighted by the dashed blue box (**Figure** **12**a). Figure 12b shows the Raman spectrum taken from the black region, three peaks positioned at around 1331, 1589, and 2650 cm^−1^ were attributed to the D, G, and G′ bands of graphene, respectively.^103^ Intensity of the D band was stronger as compared to the intensity of G band. This was attributed to the very strong interaction between carbon and Si, as carbon atoms had very low diffusivity on Si surface, and did not move as freely as on metal surfaces. Raman spectrum taken from the white region (Figure 12c) shows the vibration curve, which was due to the optical interference in the sandwich structure of SOI, and confirmed that no graphene growth took place in that region. Several black dots and lines were observed on the sample surface grown at 870 °C (Figure 12d). However, their sizes were very small as compared to the sample grown at 970 °C (Figure 12a), which indicated that the reaction just started. No graphene signal was observed in the Raman spectrum taken from a black dot (Figure 12e). However, the interference signal of the substrate was much weaker in this case, which indicated that the surface was partially modified and started to grow graphene. Again, the growth was carried out at 895 °C and more lines along with the larger black dots were observed on the surface (Figure 12f). Raman spectrum taken from a black region is shown in Figure 12g. The peaks positioned at 1331 and 1594 cm^−1^ were attributed to the D and G bands of graphene, respectively.

Furthermore, growth at 945 °C yielded larger black areas and almost fully covered the substrate surface. However, the Raman spectrum only exhibited a strong fluorescence curve in this case. This may be attributed to the fact that after strong surface reaction, the surface optical properties of SOI might have changed reasonably. They also claimed that H_2_ was not necessary to grow graphene on SOI and just replaced 6 sccm of H_2_ with 50 sccm of Ar at 920 °C. Small black dots were observed and the SOI surface seemed to be less reactive. When the growth was carried out at 970 °C, the size of the black dots increased and stronger graphene bands were observed. Based on the above results, they proposed a growth mechanism for graphene on nonmetal or semiconducting surfaces as surface reaction, adsorption, decomposition, and accumulation (Figure 12h). At high temperatures, the native oxide layer decomposed and Si surface became reactive with free dangling bonds. The surface got clean, especially under H_2_ flow by etching out SiO*_x_* species. Carbon atoms released from thermal cracking of CH_4_ were adsorbed on a clean reactive Si surface and strongly bonded with surface dangling bonds. Consequently, surface‐adsorbed carbon atoms did not move freely on Si surface and CH_4_ molecules continued to accumulate at this spot and neighboring Si atoms. Eventually, the accumulated carbon atoms combined together to form graphene.

Tai et al. obtained much better results by using APCVD system as they demonstrated direct growth of atomically flat SLG or bilayer graphene (BLG) domains, concave BLG domains, and bulging FLG domains on the upside‐down placed single crystalline Si substrates at 900–930 °C for 1 h using the composition of CH_4_ and H_2_ gases.^104^ It was observed that a higher growth temperature caused larger domain size and higher nucleation density. However, uniform‐continuous‐large area graphene films could not be achieved, as the surface of Si was damaged at higher temperatures (>950 °C). Large graphene domains on Si could be achieved with the help of trace oxygen in this temperature range as obtained on dielectric and metallic substrates.^23^, ^105^

**Figure 13:**
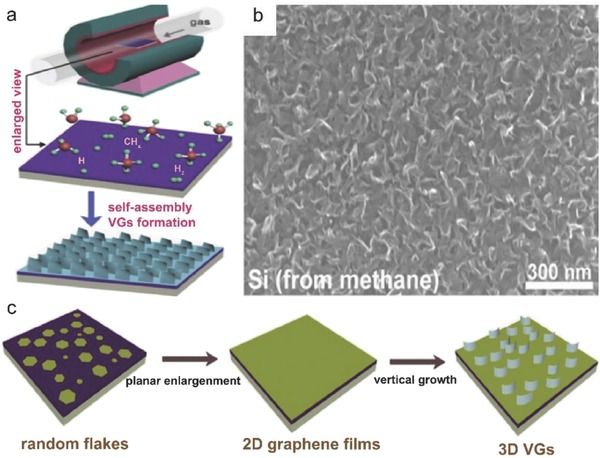
a) Schematic illustration of the template‐free and catalyst‐free CVD growth process of VAGNs. b) SEM image of VAGNs grown on Si substrate by using CH_4_ as carbon precursor. Conditions: VAGNs were grown at 8 sccm CH_4_, 50 sccm H_2_, and 50 sccm Ar for 6 h. c) Schematic illustration of time‐dependent evolution of the VAGNs growth. Reproduced with permission.^27^ Copyright 2017, Elsevier Ltd.

Recently, Wang et al. demonstrated the direct synthesis of uniform VAGNs on Si substrates via catalyst‐free thermal CVD for the first time.^27^ The VAGNs were grown using either CH_4_ or ethanol as the carbon feedstock in a conventional thermal APCVD system. It was also established that the concentration of active carbon species in CVD system exerts significant impact on the growth mode of graphene. The carbon precursor flow rate and reaction time during the CVD process controlled the growth dynamics, which ultimately led to a well‐controlled morphology of the obtained carbon material, such as 2D graphene film or VAGNs. The growth morphology was found to be independent of substrate as well as carbon source precursor. This growth strategy suggested that VAGNs can be fabricated on variety of substrates using different carbon precursors in a thermal APCVD system without using plasma, and offers a new insight for intrinsic growth mechanism. System was heated to 1130 °C in 50 min with 50 sccm H_2_ and 50 sccm Ar. The substrates were annealed for 20 min at 1130 °C, and then CH_4_ or ethanol (vapors) was introduced into the chamber to initiate graphene growth. The graphene growth morphology could be controlled by varying the precursor concentration and/or reaction time. The VAGNs growth process is schematically depicted in **Figure** **13**a. The height of VAG sheet was around 200 nm as observed from the 45° tilt side‐view image. The evolution of VAGNs was monitored by varying the growth time, and the detailed process is schematically depicted in Figure 13c.

Initially, graphene flakes were randomly nucleated on the substrate surface and enlarged to form continuous film, as time progressed. Once a buffer layer formed, initial planar graphene growth eventually altered to upward growth. Consequently, the carbon atoms from precursor thermal cracking were continuously incorporated into the open edges to make the unique vertical growth proceed. VAGNs were obtained with the CH_4_ flow rate between 7 and 14 sccm, whereas planar growth was observed for less than 7 sccm CH_4_ flow rate. The carbon precursor decomposition under low CH_4_ flow rate synchronized with the adsorption of carbon atoms on active sites and the growth of graphene. Therefore, the diffusion of carbon species to edge of graphene proceeded under a thermodynamic equilibrium condition, and had enough time to reach the desired positions with minimum energy to form stable crystalline phases.^106^ Consequently, only 2D stacked nanometer‐sized or micrometer‐sized graphene flakes and films were observed even after 10 h growth.^28^ However, at higher CH_4_ flow rates, the effective diffusion of carbon species was limited on the substrate or graphene surface. Therefore, a multiple graphene nucleation and simultaneous enlargement took place, which was induced by the supersaturation of active carbon species concentration, and finally led to the shrinking of available surface on the substrate into narrow channels. In this way, surface diffusion of carbon species reduced drastically and the direct deposition of carbon species started at the graphene edges. Hence, the edge reaction occurred very fast, and led to the vertical growth instead of boundary coalescence when two graphene domains approached each other.^107^, ^108^, ^109^

#### 3.1.3 High Temperature Growth on Textured Substrates

**Figure 14:**
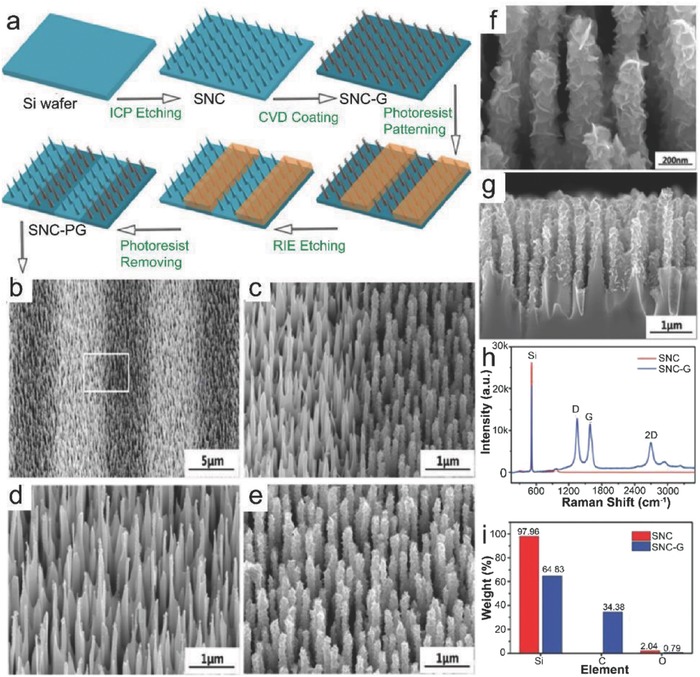
a) Schematic diagram of the fabrication process of the SNC, SNC‐G, and SNC‐PG electrodes. b) A SEM image of the SNC‐PG electrode corresponding to the schematic diagram. c) A higher magnification SEM image for the SNC‐PG electrode from the white box in (b). d) Tilted angle SEM image of the SNC electrode. e) Tilted angle, f) high‐magnification tilted angle, and g) cross‐section SEM images of the SNC‐G electrode. h) Raman spectra and i) energy dispersive X‐Ray (EDX) element weight ratios of the SNC electrode and the SNC‐G electrode. Reproduced with permission.^111^ Copyright 2017, the Royal Society of Chemistry.

Instead of using flat Si surfaces for the metal‐catalyst free direct CVD growth of graphene, researchers have used textured Si substrates to grow graphene.^110^, ^111^ Li et al.^110^ and Wang et al.^111^ reported the direct growth of VG on Si nanocones (SNCs) with the help of hot‐filament CVD (HFCVD) system for field emission and electrochemical applications, respectively. The SNC electrode was fabricated by an inductively coupled plasma (ICP) reactive ion etching system. The SNC‐graphene (SNC‐G) electrode was patterned to get SNC‐patterned G (SNC‐PG) electrode by using UV‐lithography (UVL) and reactive ion etching (RIE), which enabled the SNC structure and SNC‐G structure got tested on the same electrode. The whole fabrication process of the SNC, SNC‐G, and SNC‐PG electrodes is schematically depicted in **Figure** **14**. First, the SNC electrode was fabricated by etching the Si wafer using O_2_ and SF_6_ in an ICP reactive ion etching system. Second, the VG was directly grown on the SNCs using CH_4_, H_2_, and Ar in the ratio of 1:5:45 at 1000 °C for 3 min. The chamber pressure was maintained at 2.5 kPa, and a bias voltage was applied between the filament and the substrate to facilitate the growth of graphene. Finally, SNC‐ PG electrode was fabricated by patterning the SNC‐G electrode with the help of UVL and RIE techniques. The tilted angle SEM images of the SNC electrode and SNC‐G electrode are shown in Figure 14d,e, respectively. Whereas, high magnification tilted angle and cross‐sectional SEM images of SNC‐G electrode are shown in Figure 14f,g, respectively.

It was observed that the graphene nanosheets (GNs) were vertically grown on the SNCs and resembled to petaloid clusters. The average height and half‐width of the vertically aligned SNCs were found to be 2 µm and 100 nm, respectively, and the average half width of the SNCs with VG coating was 200 nm. The GNs grew vertically after covering the SNCs surface and formed 3D petaloid structures due to the internal stress and the applied bias voltage.^110^ Raman spectra of the SNC electrode as well as the SNC‐G electrode are shown in Figure 14h. The peak positioned at ≈520 cm^−1^ belongs to Si, whereas the characteristic peaks of few‐layered graphene marked as D, G, and 2D were observed for the SNC‐G electrode.^112^ Elemental analyses of the SNC electrode and the SNC‐G electrode are shown in Figure 14i. For the SNC electrode, carbon was absent while the presence of a small amount of O_2_ (2.04%) was due to the surface oxidation. The SNC‐G electrode contained carbon along with Si, and a very small amount of O_2_ (0.79%). Similarly, Son et al.^113^ demonstrated the direct growth of high quality MLG on Si nanoparticles at 900–1100 °C via APCVD using a gas mixture of CH_4_, CO_2_, and H_2_. CO_2_ was used as a mild oxidant which helped in achieving robust and uniform growth of MLG around each Si nanoparticle by generating catalytic sites.

### 3.2 Catalyst‐Free Direct CVD Growth of Graphene on Ge Substrates

**Figure 15:**
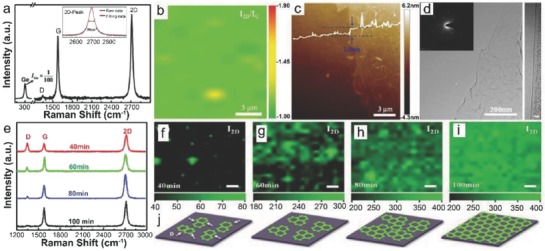
Large‐scale uniform growth of monolayered graphene films on Ge substrate. a) Raman spectrum of graphene on Ge substrate. The inset shows the FWHM and the Lorentzian fitting of 2D peak. b) 2D Raman mapping of the *I*_2D_/*I*_G_ peak intensity ratio obtained from the graphene deposited on Ge (15 µm × 15 µm region with the step size of 1 µm). c) Contact‐mode AFM image of a graphene film transferred on SiO_2_ showing the monolayered feature and wrinkles. d) TEM image and SAED pattern revealing the high crystalline quality of the graphene and HR‐TEM image showing that the graphene is monolayered. The scale bar in the HR‐TEM image is 3 nm. Characterization of graphene grown on Ge substrates for different durations and illustration of graphene growth evolution. e) Raman spectra of graphene films deposited on Ge under optimal conditions for different time. f–i) Color‐coded Raman mapping of the 2D peak intensity images of graphene as a function of deposition time. The green features are graphene domains and the dark regions represent the bare Ge surface. The scale bar is 2 µm. j) Schematic illustration of evolution of the graphene films on Ge for different deposition time. Reproduced with permission.^18^ Copyright 2013, Springer Nature.

As discussed earlier that Ge possesses higher catalytic ability, very low carbon solubility, and high diffusivity even at its melting point as as compared to Si. Thus, immiscible Ge‐C system under equilibrium conditions dictates graphene growth on Ge via self‐limiting and surface‐mediated process instead of precipitation process as observed for metals with high carbon solubility. Wang et al. reported the direct growth of single‐layered graphene on Ge substrate via APCVD, and it was a large‐area and uniformly deposited high‐quality graphene.^18^ Generally, the amount of hydrocarbon gas and H_2_ determines the number of grown graphene layers, as H_2_ balances the production of reactive hydrocarbon radicals and etching of graphitic carbon during a CVD process. Therefore, the optimized graphene growth was carried out with H_2_:CH_4_ = 50:0.1 sccm at 910 °C for 100 min to obtain single‐layered graphene. **Figure** **15**a shows the Raman spectrum of the as‐grown graphene with very weak intensity of the D band, which indicated that the grown graphene film was of high quality similar to exfoliated graphene.^114^ The symmetric 2D peak with a FWHM of ≈30 cm^−1^ (inset Figure 15a) was well fitted by a single Lorentzian curve, which confirmed the growth of single‐layered graphene.^115^ Raman mapping of the 2D to G peak intensity ratio over a 15 µm × 15 µm area with a spot size of 1 µm and a step size of 1 µm was carried out, which revealed that the *I*_2D_/*I*_G_ ratio was quite uniform over the region studied (Figure 15b). The *I*_2D_/*I*_G_ was in the range of 1–1.5, which confirmed the complete SLG coverage.^116^ The AFM micrograph of the transferred graphene film from Ge onto 300 nm SiO_2_/Si substrate with a uniform height of 1.1 nm (Figure 15c) also confirmed that the graphene film was single‐layered.^117^ The single‐layer and single‐crystalline nature of the grown graphene was also confirmed by TEM and SAED analyses as shown in Figure 15d. Furthermore, it was also observed from the Raman spectra (Figure 15e) that the D peak disappeared gradually as the growth time reached to 100 min. Similar results were obtained for the samples grown for 120 min or longer durations, which indicated that the growth on Ge was self‐limited. Figure 15f–i shows the investigation of graphene domains expansion by the color‐coded intensity mapping of the 2D peak over an area of 15 × 15 µm^2^ with a spot size of 1 µm and step size of 1 µm. The green and the dark regions correspond to graphene domains and bare Ge surface, respectively. Initially, the size of the graphene domains was relatively small and there were a large number of edge defects related to the domains of graphene, which led to the remarkable D peak in the Raman spectra. As the growth time reached to 100 min, the graphene domains grew in 2D islands due to excess carbon atoms, and finally merged together to form a continuous film (Figure 15j). The constituents in the Ge–C alloy were immiscible under equilibrium in the bulk according to equilibrium phase diagram of the Ge–C system and resembled to the Cu–C system, which is known to be mutually immiscible in the solid and liquid states.^118^ Moreover, the properties of the graphene films grown on Ge were the same irrespective of fast or slow cooling process. As the carbon solubility in bulk Ge (<0.1 atm %) is negligible, therefore a self‐limiting and surface‐mediated growth process was observed similar to Cu‐catalyzed growth of graphene.

**Figure 16:**
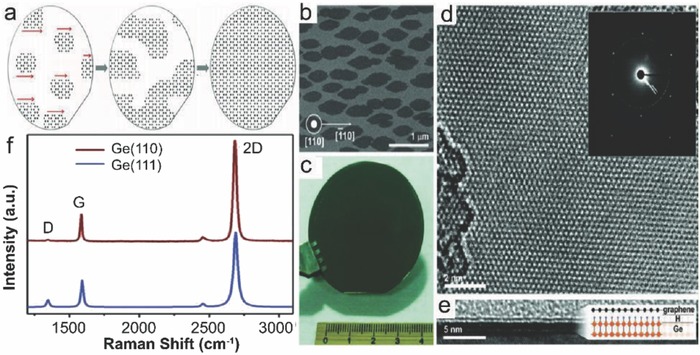
Single‐crystal monolayer graphene grown on a hydrogen‐terminated Ge(110) surface. a) A schematic illustration of catalytic growth of single‐crystal monolayer graphene from unidirectionally aligned multiple seeds. b) A typical SEM image of graphene seeds at the early stage of growth. c) A photograph of graphene grown on a 5.08 cm Ge/Si (110) wafer. d) A HRTEM image of the single‐crystal monolayer graphene. Inset: Four overlaid SAED patterns, which were measured across the four different points. The distance between each point is ≈2 mm. e) A cross‐sectional TEM image demonstrating that the as‐grown graphene is monolayer. Inset: A schematic illustration of the monolayer graphene grown on the H‐terminated Ge surface. f) Raman spectra of single‐crystal and polycrystalline graphene grown on H‐Ge(110) and H‐Ge(111) surfaces under same growth conditions. a.u.: arbitrary units. Reproduced with permission.^14^ Copyright 2014, American Association for the Advancement of Science.

Catalytic growth of a single‐crystalline graphene on a solid substrate surface can be achieved by growing a single grain to a size as large as possible from a single nucleation site. Recently, a centimeter‐sized single‐crystalline graphene was obtained from a single nucleus.^105^ Another way is to grow graphene on a single‐crystalline substrate where multiple nucleations of the graphene domains could take place with perfect rotational alignment. Finally, these unidirectionally aligned domains grow and coalesce to form a uniform single‐crystalline graphene without grain boundary defects (**Figure** **16**a).^14^ Lee et al. demonstrated a wafer‐scale growth of wrinkle‐free single‐crystal monolayer graphene on the reusable hydrogen‐terminated Ge(110) and Ge (111) buffer layers supported on Si.^14^ The Ge (110) surface's anisotropic twofold in‐plane symmetry enabled unidirectional alignment and coalescence of multiple seeds to form uniform single‐crystal graphene with predefined orientation. Moreover, the weak interaction between graphene and hydrogen‐terminated Ge surface helped in etch‐free dry transfer of graphene, and the reuse of the Ge substrate for continual graphene growth. Highly uniform graphene monolayers were grown on hydrogen‐terminated Ge surfaces on Si (110) via LPCVD by flowing CH_4_ gas (1–2% diluted in H_2_) at 900–930 °C for 5–120 min. Initially, the graphene islands were uniaxially aligned along the^110^ direction of the underlying Ge (110) surface (Figure 16b), and eventually formed uniform monolayer graphene on the whole substrate (Figure 16c). A HRTEM image (Figure 16d) confirmed the formation of monolayer graphene without any noticeable structural defects. The overlaid SAED patterns acquired from four different points separated from each other by ≈2 mm confirmed that all of the points were having the same crystallographic orientations (Figure 16d, inset). Moreover, the cross‐sectional TEM image also confirmed that the as‐grown graphene was monolayered (Figure 16e). Similar results were also obtained for graphene growth on an isotropic Ge (111) surface, but the grown graphene was polycrystalline in nature. Raman spectra of the grown samples also confirmed that both materials were monolayer graphene (Figure 16f).^119^

However, *I*_D_/*I*_G_ ratio of the single‐crystal graphene (<0.03) was much smaller than that of the polycrystalline graphene (≈0.4), which indicated that the extended grain boundary defects in the single‐crystal graphene on Ge (110) were absent. Hence, it can be inferred that the hydrogen‐terminated Ge (110) surfaces are an ideal substrate for the catalytic growth of single‐crystalline monolayer graphene. On the other hand, Dai et al. also demonstrated the metal‐catalyst free direct CVD growth of graphene on Ge (110) wafers instead of Ge (110) buffer layers on Si (110).^120^ On the basis of experimental and theoretical investigation, they established that the lattice matching phenomenon between atomic steps on the Ge (110) surface and graphene edges were mainly responsible for the unidirectional alignment of graphene islands. Graphene islands were attached to the atomic steps of the Ge (110) surface by strong chemical bonds with their armchair directions along the [−110] direction of the Ge (110) substrate.

After the successful direct CVD growth of single‐crystalline and polycrystalline graphene monolayer, the direct CVD growth of graphene nanoribbons (GNRs) on Ge was reported by Jacobberger et al. for the first time.^121^ They proposed that the transition of graphene from a semimetal to a semiconductor could be possible if it is confined into nanoribbons (NRs) narrower than 10 nm with controlled crystallographic orientation, and well defined armchair edges. GNRs were grown on Ge (001) via APCVD. Ge (001) (Wafer World, resistivity >40 Ω cm, miscut <1°) substrates were successively cleaned with acetone and isopropyl alcohol for 15 min followed by etching in deionized H_2_O (18 MΩ cm) at 90 °C for 15 min. The cleaned substrates were loaded into a horizontal tube and the system was evacuated to ≈10^−6^ Torr. It was filled to atmospheric pressure with a mixture of Ar (99.999%) and H_2_ (99.999%) at a constant total flow rate of 300 sccm. The Ge (001) samples were annealed for 30 min at 910 °C, and then CH_4_ (99.99%) was introduced to initiate the growth for 1–18.25 h. The GNRs were found to be self‐aligned 3° from the Ge 〈110〉 directions, and self‐defined with smooth armchair edges. They were having tunable width to <10 nm and aspect ratio to >70. It was also observed that, to obtain highly anisotropic GNRs, the growth rate in the width direction should be very slow (<5 nm h^−1^).

It was inferred that the energy barrier associated with the graphene nucleus rotation significantly increased, as it became larger, which resulted in fixing the orientation of the NR lattice during the subsequent growth.^122^ As the Ge (001) surface consists of two types of terraces having the same structure but rotated 90° with respect to each other, therefore it might be possible that on one set of terraces, the armchair direction of the graphene nuclei was rotated 3° from Ge,^110^ whereas on the other set it was rotated 3° from Ge [−110]. The anisotropic growth was due to preferential attachment of intermediate hydrocarbons from the Ge surface to the short (faster growing) ribbon edges over the long (slower growing) ribbon edges. Therefore, low aspect ratio crystals and the NRs had different lattice orientation. This growth method allows successful fabrication GNRs directly on technologically important semiconducting substrates for future electronic applications.

Kiraly et al. studied the metal‐catalyst free direct CVD growth of graphene on differently oriented Ge substrates in order to understand the nature of the graphene–Ge interfaces.^123^ Graphene samples were directly grown on Ge (001), Ge (110), and Ge (111) substrates via APCVD at 910 °C by fixing the flow rates of CH_4_, H_2_, and Ar at 3.6–4.6, 100, and 200 sccm, respectively. After ultra high vacuum (UHV) annealing to 700 °C, both the Ge (110) and the Ge (111) surfaces restructured into domains, and exhibited in‐plane ordering underneath. The Ge(111)/graphene interface was strongly affected by UHV annealing. STS analyses revealed significant differences in electronic interactions between graphene and Ge (110)/Ge (111). Raman spectra indicated that the graphene was considerably strained after the growth with more point‐to‐point variation especially on Ge (111). Finally, the extreme strained case was observed for graphene/Ge (001), which resulted in the reorganization of the Ge surface into^107^ facets. Upon UHV annealing, the native strain influenced the atomic structure of the interface by inducing metastable and previously unobserved Ge surface reconstructions. These nonequilibrium reconstructions covered almost more than 90% of the surface, which could modify both the electronic and mechanical properties of the graphene overlayer.

**Figure 17:**
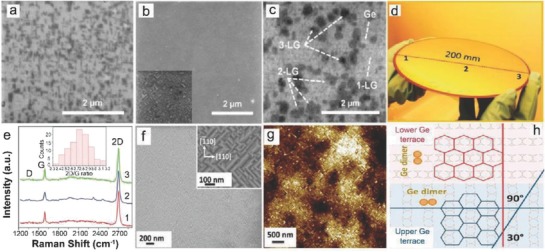
SEM images of samples grown at different CH_4_ flows *F* for *t*_D_ = 60 min. a) *F* = 1 sccm; b) *F* = 2 sccm; and c) *F* = 5 sccm. Inset in panel (b) reports the SEM image of the sample at *F* = 2 sccm acquired at higher magnification. The panel and its inset have the same scale bar. In panel (c), the arrows mark regions having different number of graphene layers that appear with different grayscale intensity. Reproduced with permission.^126^ Copyright 2016, American Chemical Society. d) Graphene grown on 200 mm Ge/Si wafer and e) Raman spectra at the indicated places. The histogram of the 2D/G ratio over the entire wafer (≈100 measured points) is depicted in the inset of panel (e). f) SEM and g) AFM images of the graphene on Ge(001). Reproduced with permission.^130^ Copyright 2016, American Chemical Society. h) The presence of two orientational domains of graphene on Ge(100) indicates that the growth of graphene is correlated with the direction of Ge dimer rows. Reproduced with permission.^131^ Copyright 2017, The Electrochemical Society.

Despite the fact that the direct CVD growth of graphene on Ge (100) results in highly strained graphene overlayers, Pasternak et al. demonstrated the direct LPCVD growth of large area high‐quality graphene films on Ge (100) overlayers supported on Si (100) substrates by using CH_4_ gas at 900–930 °C^124^, ^125^ . After this, Scaparro et al. attempted the direct CVD growth of graphene on Ge(100) substrates by varying the H_2_/CH_4_ flow ratio, and the growth time in order to understand the growth mechanism and morphology.^126^ Graphene samples were grown in a commercially available 4 inch cold‐wall LPCVD reactor with 200 and 800 sccm flow rates of H_2_ and Ar, respectively, whereas the CH_4_ flow rate varied between 1 and 10 sccm. Once the growth temperature reached to 930 °C, CH_4_ was introduced into the chamber, and the total pressure was set to 100 mbar. It was possible to tune the growth in order to obtain different graphene structures, such as GNRs, monolayered and multilayered graphenes by simply varying the CH_4_ flow and growth time. At a flow rate of CH_4_ (*F*) = 1 sccm, GNRs oriented along the 〈110〉 directions on the surface (**Figure** **17**a) similar to as discussed earlier.^121^ The asymmetry in their shape (high length/width aspect ratio) was due to the Ge (100) surface anisotropy.^127^ At *F* = 2 sccm, a uniform SLG film without domains was obtained (Figure 17b), whereas the inset shows the nanotextured Ge surface underneath. The Ge nanofaceting appeared only when a continuous or a quasicontinuous graphene film was obtained. This was attributed to the development of local strain of the Ge surface induced by the growth of large enough and ordered graphene domains. Similarly, at *F* = 5 sccm, single, bilayer, and trilayer graphene domains were observed on the Ge surface (Figure 17c). The increase of carbon adatom species (CH*_x_*) concentration led to strong supersaturation. The formation of carbon growth species was much faster as compared to their total consumption, which allowed the further nucleation of graphene domains.^128^ The growth mode observed here was also mentioned by Pasternak et al.^124^, ^125^ Furthermore, at higher flow rates of CH_4_, poorer quality graphene films with absence of the Ge nanofaceting were obtained. The time dependent growth at *F* = 2 sccm and H_2_/CH_4_ = 100 for 60 min was carried out several times. The growth proceeded in a layer‐by‐layer regime, as the carbon adatom species (CH*_x_*) concentration before nucleation was just above the critical supersaturation level. The adsorbed carbon species depleted as the nucleation and growth of graphene grains started. Eventually, their concentration was reduced to a level where the nucleation rate could be negligible and only monolayered domain enlargement could take place. Moreover, the initial supersaturation condition was restored and the second graphene layer formation started to take place. These results can be attributed to the similarities between the C–Cu and C–Ge alloy systems.^18^, ^128^, ^129^

In continuation to the metal‐catalyst free direct CVD growth of graphene on Ge substrates, Lukosius et al. and Lupina et al. reported a complementary metal oxide semiconductor (CMOS) technology compatible good quality graphene on 200 mm Ge(001)/Si(001) wafers, respectively.^130^, ^131^ Dabrowski et al. presented a detailed understanding of direct CVD growth mechanism of graphene on Ge (001)/Si (001) substrates.^132^ Prior to the graphene growth, 2 µm thick Ge (001) layers were grown on 200 mm Si (001) wafers by CVD.^130^, ^133^ Thereafter, graphene growth was carried out at 885 °C for 60 min using CH_4_ and Ar/H_2_ mixtures, while the total system pressure was maintained at 700 mbar.

In order to investigate the quality of the grown graphene on 200 mm wafer, several Raman spectra were obtained. Only three of them are shown in Figure 17e, which were taken from the selected areas indicated as 1, 2, and 3 in Figure 17d. The grown graphene was having very small *I*_D_/*I*_G_ ratio (≈0.1), which was the indication of a low concentration of defects. Thus, a good quality, uniform, and large area graphene was obatined. Graphene‐induced Ge faceting was also observed in this case (Figure 17f,g), as observed and discussed previously.^125^, ^134^ The typical heights of the facets and *R*_a_ values were found to be 2–5 nm and 0.7–1.1 nm, respectively [from AFM image (Figure 17g)]. Generally, the majority of crystallites are oriented either parallel or perpendicular to the (110) axis in the CVD graphene on Ge (001), where the carbon hexagons have sides either parallel or perpendicular to the surface dimer rows (Figure 17h).^121^, ^123^, ^131^, ^132^ It could be possible that the small graphene grains with the same orientation could coalesce into larger strained grains, however their sizes will be limited by the distance between surface steps, as the growth direction rotates by 90° from terrace to terrace. Two oriented grains cannot coalesce into a single one (Figure 17h), as the terrace width on a nominally flat surface can hardly exceed about 100 nm.

Dabrowski et al.^132^ suggested that the growth of large graphene grains on Ge(100)/Si (100) could be achieved by suppressing the nucleation at most of the potential nucleation sites with the help of H_2_, whereas increased H_2_ coverage may reduce growth rate and hence could increase growth time. Consequently, surface segregation of Si could increase and may result in surface carbide formation, whereas it was already demonstrated that unidirectional‐oriented islands coalesced to form single‐crystalline graphene on Ge (110) surfaces without grain boundary defects.^14^, ^120^ Recently, Jacobberger et al. again demonstrated the direct APCVD growth of semiconducting armchair graphene NRs on Ge (001) wafers as demonstrated earlier. GNRs were transferred onto SiO_2_/Si or HfO_2_/Si wafers to fabricate FETs.^121^ As discussed earlier that GNRs as narrow as 2 nm were obtained, and the edges consisted of smooth armchair segments.^121^, ^127^ The GNRs growth was carried out for 2 h at 910 °C with 2 sccm CH_4_ and rest of the process was same as described in ref. 121. It was observed that arrays of randomly distributed GNRs were aligned roughly along the Ge〈110〉. Using a dry transfer method,^105^ the GNRs were transferred onto SiO_2_ (15 nm)/Si or HfO_2_ (15 nm)/Si substrates to fabricate FETs.

**Figure 18:**
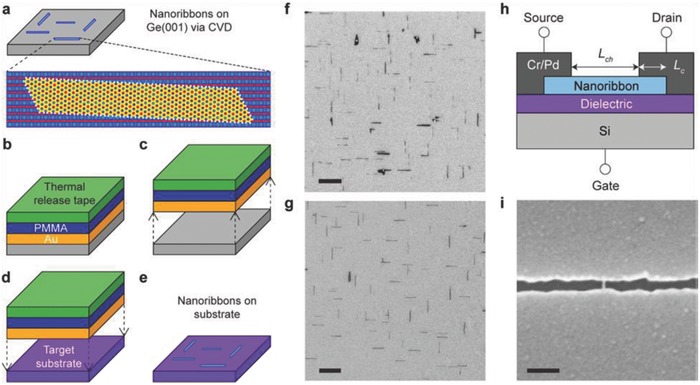
Schematic of the nanoribbon transfer. a) Graphene nanoribbons with predominately smooth armchair edges and that are aligned roughly along Ge〈110〉 are grown on Ge(001) via CVD. b) The ribbons are coated with 60 nm of Au, 300 nm of PMMA, and thermal release tape. c) The thermal release tape is lifted to separate the ribbons from Ge(001), and d) the ribbon array is stamped onto the target substrate. e) The thermal release tape is released by applying heat, followed by removal of PMMA in acetone and etching of Au in KI/I_2_/H_2_O. SEM images of nanoribbons f) after growth on Ge(001) and g) after transfer to SiO_2_. h) Schematic of the nanoribbon FET architecture in which the nanoribbon channel (with channel length of *L*_ch_) is contacted by Cr/Pd/Au source and drain electrodes (with a contact length of *L*_c_), the Si substrate serves as the back gate, and SiO_2_ or HfO_2_ serves as the gate dielectric. i) SEM image of an FET with nanoribbon channel with apparent width of ≈7 nm and *L*_ch_ of ≈25 nm. Scale bars in (f), (g), and (i) are 1 µm, 1 µm, and 100 nm, respectively. Reproduced with permission.^135^ Copyright 2017, American Chemical Society.

The GNRs were peeled off with the help of a sacrificial multilayer stack of Au/poly(methyl methacrylate) (PMMA)/thermal release tape, and stamped onto the desired substrate (**Figure** **18**a–e). No changes in the alignment and position of the GNRs were observed before (Figure 18f) and after (Figure 18g) transfer. Source and drain electrodes with channel length (*L*_ch_) of 25–120 nm were defined via e‐beam lithography. Cr/Pd/Au (0.7/10/8.3 nm) contacts were thermally evaporated and patterned at random locations across the substrate so that the ribbons could be perpendicular to the source and drain electrodes. A schematic diagram of the FET architecture is shown in Figure 18h. Figure 18i shows a SEM image of a FET with a GNR channel with apparent width of ≈7 nm and *L*_ch_ of ≈25 nm. The direct CVD growth of aligned, narrow, and semiconducting GNRs on Ge (001) could overcome many challenges, like control over the polydispersity in ribbon width, length, and location. Thus, it may enable significant advances in state‐of‐the‐art semiconductor electronics.

### 3.3 Catalyst‐Free Direct CVD Growth of Graphene on Important Wide Bandgap Semiconducting (GaN and SiC) Substrates

Silicon carbide (SiC) and gallium nitride (GaN) are two important wide bandgap semiconductors which can be integrated with graphene for electronic and optoelectronic device applications. There are only few reports available on the metal‐catalyst free direct CVD growth of graphene on SiC and GaN substrates. Unfortunately, no reports are available on direct CVD growth of graphene on other wide bandgap semiconductors. Direct CVD grown graphene‐based coatings as transparent electrodes can be utilized in optoelectronics, especially for fabricating GaN‐based light emitting diodes. So far, this has been achieved by transferring CVD grown graphene from metal substrates to GaN surface.^136^, ^137^, ^138^ For the first time, Sun et al. reported direct CVD growth of graphene‐like large‐area carbon thin films on GaN.^139^ The grown carbon thin films were transparent and conducting, wheras the quality was inferior as compared to standard graphene. Unintentionally doped 3.5 µm thick GaN (0001) was grown on sapphire by metal organic CVD (MOCVD). Carbon thin films were grown at 950 °C by flowing 160 sccm C_2_H_2_ and 1000 sccm NH_3_ for 5 min, while the total pressure was maintained at 750 mbar. As GaN can dissociate at high temperatures, an overpressure of NH_3_ was maintained to protect the GaN surface. NH_3_ plays a dual role, as it compensates the loss of nitrogen from GaN during growth as well as releases H_2_, which is helpful in graphene‐CVD process. The thickness of the grown carbon thin films was in the range of 2–4 nm and they were continuous, uniform, and scalable.

**Figure 19:**
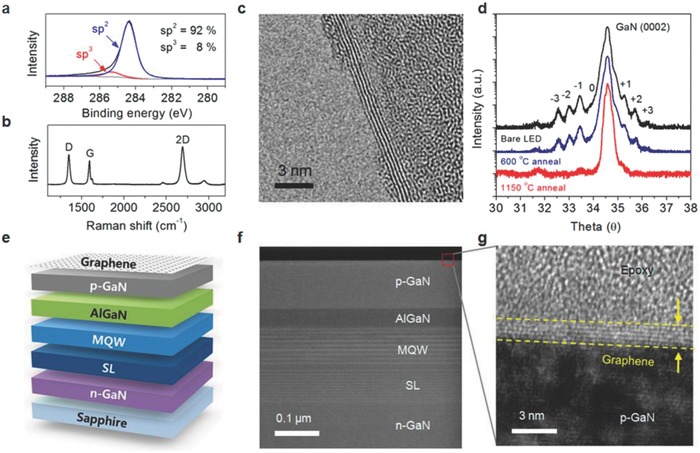
a) XPS spectrum of the C 1s core level and b) Raman spectrum of a DG film synthesized for 3 h under a plasma power of 50 W. c) HRTEM image of the graphene edge on a TEM grid. d) HR‐XRD curves of the InGaN/GaN MQWs before and after thermal treatment. e) Schematic diagram and f) scanning TEM image of the DG/LED structure. g) HRTEM image at the interface between the p‐GaN and DG electrodes. Reproduced with permission.^140^ Copyright 2014, American Chemical Society.

Since PECVD is a low temperature process, therefore direct growth of uniform graphene films on GaN via PECVD seems to be an ideal option to fabricate optoelectronic devices in order to prevent thermal degradation of GaN and other active layers. Kim et al. demonstrated the direct integration of polycrystalline graphene into GaN‐based light emitting diodes (LEDs) via PECVD.^140^ Graphene films were grown at 600 °C for 1–3 h by flowing a mixture of CH_4_ (2 sccm) and H_2_ (20 sccm) with the total pressure maintained at 10 mTorr. The power for discharging the gas mixture was 50 W, and CH_4_ was effectively dissociated into various species, such as CH*_x_*, C_2_H*_y_*, H, and H_2_. The dissociation rate was about 34%, which was much higher as compared to ≈0.0002% in thermal CVD,^141^ which indicated that the plasma assistance effectively reduced the activation energy for the direct growth of graphene at 600 °C as compared to the graphene growth (*E*_a_ ≈ 2.0–2.6 eV) in thermal CVD.^128^, ^142^ The main peak at 284.4 eV (≈92%) was the characteristic signal of sp^2^‐hybridized carbon atoms in as‐grown graphene (**Figure** **19**a). A typical Raman spectrum of the grown graphene film is shown in Figure 19b. The peaks D (≈1350 cm^−1^), G (≈1580 cm^−1^) and 2D (≈2680 cm^−1^) confirmed the formation of graphene film with structural disorder. Figure 19c is the HRTEM image which confirmed the layered structure and polycrystalline nature of the graphene film. The XRD patterns of a bare LED and the annealed LEDs at 600 and 1150 °C are shown in Figure 19d, respectively. The X‐Ray diffraction (XRD) patterns of the bare LED, and that was annealed at 600 °C, were found to be identical, which indicated that the thermal degradation of the InGaN/GaN multiquantum well (MQW) layers was negligible at 600 °C growth temperature of graphene in the PECVD system.

Hence, GaN‐based blue LEDs were fabricated with directly grown graphene through a batch process for centimeter‐scale LED substrates, and their schematic diagram and a scanning TEM image with a graphene electrode are shown in Figure 19e,f, respectively. High‐magnification image of the interface of the graphene and p‐GaN is shown in Figure 19g, which confirmed the layered structure of directly grown graphene on the p‐GaN surface. Additionally, in situ ohmic contact formation was observed between the directly grown graphene and p‐GaN as carbon diffused into the p‐GaN surface during the growth process. Therefore, the contact resistance was also reduced, and these directly integrated LEDs exhibited superior electrical properties as compared to the LEDs fabricated with transferred graphene electrodes.

Similarly, low‐temperature direct PECVD growth of island type nanographene and vertical graphene nanosheets on SiC substrates using CH_4_ were demonstrated by Zhang et al. and Ghosh et al., respectively.^24^, ^143^ The quality of graphene produced by them was not good, and the detailed study investigating how the growth parameters affected the morphology and mechanism was not carried out as well. Recently, high‐temperature direct CVD growth of high quality monolayer and few layers graphene on 6H‐SiC (0001) substrates was reported by Yang et al.^144^ The growth was carried out in a rapid‐heating (RH)‐LPCVD at 1250–1550 °C by utilizing a gas mixture of Ar (10–50 sccm), H_2_ (4–32 sccm), and CH_4_ (0.5–8 sccm) for 1–6 min. Before each sample growth, substrates were heated to 1600 °C for 3 min under a 100 mbar pressure to sublimate the Si from top of the SiC surface, which exposed the inner carbon and provided the spontaneous nucleation site for external carbon species. All the important results on low and high temperature metal‐catalyst free direct CVD growth of graphene on Si, Ge, GaN, and SiC are summarized in **Table** **2**.

**Table 2:** Summary of the catalyst‐free direct CVD growth parameters and morphology of graphene on semiconductors

Substrate	CVD systems	Precursors (solid/liquid/gas)	Flow rate of Ar/H_2_ [sccm]	Time [min]	Temp [°C]	Morphology	Ref.
Si (001)	Photoemission‐assisted PECVD (2000–4000 Pa)	CH_4_ (0.5 sccm)	Ar (1.7)	20	700	Networked nanographite	97
Si	Remote PECVD (Power: 100 W)	CH_4_ (0.204 Torr, 30 sccm)	–	180 (3 h)	525	Island type nanographene	24
Si	Microwave surface wave plasma CVD	C_2_H_2_ (5 sccm)	Ar (100) and H_2_ (0–35)	4	500	Multilayered graphene (MLG)	98
N‐Si (100)	PECVD (40 Pa)	CH_4_ (:3)	H_2_ (:4)	20–50	780	Graphene nanowalls	99
Si (100)	Thermal LPCVD (2–100 Torr)	C_2_H_2_ (25 sccm)	Ar (50)	60	800–1100	Triangle nanographenes (TNGs)	89
Si on insulator (SOI)	LPCVD (300 mTorr)	CH_4_ (35 sccm)	6 (H_2_/Ar)	30	870–970	Few layered graphene (FLG)	15
N & P type Si (100), (111), and (110)	Thermal APCVD	CH_4_ (180 sccm)	10 (H_2_)	60	900–930	SLG, BLG, and FLG	104
Si	Thermal APCVD	CH_4_ (8 sccm)	50 (H_2_/Ar)	360 (6 h)	1130	Vertically aligned GNs	27
Si	HFCVD (2 kPa)	CH_4_ (:1)	(Ar:H_2_40:10)	5–60	700–1000	Petaloid GNs on Si nanocones	110
Si	HFCVD	CH_4_ (ratio 1)	5:45 (H_2_:Ar)	3	1000	Vertical GNs on Si nanocones	111
Si nanoparticles	APCVD	CH_4_ (50 sccm) and CO_2_(50 sccm)	50 (H_2_)	10 and 20	900–1100	MLG	113
Ge	APCVD	H_2_:CH_4_ (50:0.1)	50 (H_2_)	100	910	Monolayered graphene	18
Ge (110)/Si (110) and Ge (111)/Si (110)	LPCVD	CH_4_	H_2_	5–120	900–930	Monolayered graphene	14
Ge (001)	APCVD	CH_4_ (1–4.4)	300 Ar (200) and H_2_ (100)	1–18.25	910	Armchair graphene nanoribbons	121
Ge (001), Ge (110), and Ge (111)	APCVD	CH_4_ (3.6–4.6)	Ar (200) and H_2_ (100)	–	910	Epitaxial strained graphene	123
Ge (100)/Si (100)	LPCVD (700–780 mbar)	CH_4_ (5–15)	Ar and H_2_ (20:1)	20–75	900–930	Flakes/1/2/3MLG	124
Ge (110)	APCVD	CH_4_ (0.5)	(200)Ar and H_2_ (25–30)	60–200	910	Unidirectionally aligned islands	120
Ge (100)/Si (100)	LPCVD (850 mbar)	CH_4_ 1:Ar (200)	Ar	–	900	Large area high quality graphene	125
Ge (001)/Si (001)	UHV‐CVD	C_2_H_4_ (5)	–	90–200	930	MLG	132
Ge (100)	Cold‐wall LPCVD (100 mbar)	CH_4_ (1–10)	800 and 200 (Ar and H_2_)	30–120	930	Nanoribbons/SLG/MLG	126
2 µm Ge (001)/Si (001)	LPCVD (700 mbar)	CH_4_	Ar and H_2_	60	885	Large area uniform (200 mm) MLG	130
2 µm Ge (100)/Si (100)	LPCVD	CH_4_	H_2_	60	900	High quality large area (200 mm) graphene	131
Ge (001)	APCVD	CH_4_ (2 sccm)	200 and 100 (Ar and H_2_)	120	910	Semiconducting armchair graphene nanoribbons	135
GaN (0001)	LPCVD (750 mbar)	C_2_H_2_ (158 and 160 sccm)	NH_3_ (1000 sccm)	5	950	Large area smooth and transparent carbon films	139
p‐GaN	PECVD (10 mTorr) 50 W	CH_4_ (2 sccm)	H_2_ (20 sccm)	60–180	600	Polycrystalline transparent graphene films	140
SiC	Remote PECVD (Power: 100 W)	CH_4_ (0.20 Torr, 30 sccm)	–	120 (2 h)	500	Island type nanographene	24
SiC	ECR‐PECVD (Power: 100 W)	CH_4_ (5 sccm)	Ar (25 sccm)	30	600–800	Vertical GNs	143
6H‐SiC (0001)	RH‐LPCVD (100–800 mbar)	CH_4_ (0.5–8 sccm)	Ar (10–50 sccm) and H_2_ (4–32 sccm)	1–6	1250–1550	Monolayer graphene and FLG	144

## 4 Conclusions and Outlook

Recent progress made toward the metal‐catalyst free direct CVD growth of graphene on various dielectric and semiconducting substrates is summarized in this review. The focus was on the metal‐catalyst free direct CVD growth of graphene on technologically important dielectric substrates, such as SiO_2_, ZrO_2_, HfO_2_, h‐BN, Al_2_O_3_, Si_3_N_4_, quartz, MgO, SrTiO_3,_ TiO_2_, etc., and semiconducting substrates, such as Si, Ge, GaN, and SiC. It was observed that direct CVD growth of graphene on dielectric substrates is difficult to achieve because of their low surface energy. Also, it is difficult to obtain good quality uniform graphene films on dielectric substrates via high‐temperature CVD methods. However, a low‐temperature PECVD technique could solve this problem. Apart from Ge, there are limited reports available on the metal‐catalyst free direct CVD growth of graphene on other important semiconducting substrates including wide bandgap semiconductors, such as Si, GaN, and SiC. Extremely low carbon diffusivity on Si surface and relatively high carbon solubility at high temperatures could hamper direct CVD growth of high‐quality monolayer graphene on Si substrates. Less low‐temperature direct CVD growth of graphene films on Si using different PECVD systems was attempted. However, the quality of grown graphene was not good, and large area uniform films could not be obtained. These PECVD methods mostly yielded island type growth and vertical graphene nanosheets (VGNs). The temperature of Si substrate should be lower than 1000 °C for a high‐temperature direct CVD growth of graphene. The high temperature (>800 °C) direct growth on Si substrates was attempted by using thermal APCVD, LPCVD, and HFCVD, respectively. These methods also yielded different morphologies of graphene, such as triangular nanographene, SLG, and FLG domains as well as VGNs. VGNs were grown on flat and textured Si substrates, respectively, but high quality and large‐area uniform graphene films could not be obtained. On the other hand, Ge substrate is far better than Si substrate due to its high catalytic activity and surface diffusivity, and a very low carbon solubility at its melting point (<108 atoms per cm^3^). Hence, direct CVD growth of graphene on large Ge wafers was carried out by using thermal CVD. Large area, high quality, and uniform graphene films were successfully produced. Single‐crystalline Ge substrates are also available for direct CVD growth of single‐crystalline monolayer graphene to fabricate graphene/semiconductor heterostructures based electronic devices. Furthermore, epitaxially grown large‐area single‐crystalline Ge layers on Si wafers are also available for direct CVD growth of graphene. Moreover, direct growth of uniform graphene films on GaN via PECVD seems to be an ideal option to fabricate optoelectronic devices in order to prevent thermal degradation of GaN and other active layers. As GaN can dissociate at high temperatures, therefore an overpressure of NH_3_ must be maintained to protect the GaN surface. NH_3_ plays a dual role and compensates the loss of nitrogen from GaN during growth as well as releases H_2_, which could be helpful in high‐temperature graphene‐CVD process. Direct CVD growth of graphene on Si and wide bandgap semiconducting substrates further needs to be explored to directly integrate graphene on these substrates for graphene/semiconductor based hybrid electronic and optoelectronic device applications. Attempts could be made toward growing high quality and large area graphene films on these substrates (especially Si substrates) by exploring new carbon precursors, and by designing novel CVD units. These growth methods exhibit great potentials pertaining to technology and scientific aspects, as some of the facile growth techniques have already been adopted. A detailed understanding of the metal‐catalyst free direct CVD growth mechanism of graphene on dielectric and semiconducting substrates is pivotal to achieve a controlled graphene growth on them, which is crucial for graphene based electronic and optoelectronic device fabrication.

## Conflict of Interest

The authors declare no conflict of interest.
